# Hyperbolic Surface Exciton Polaritons and Surface Waves in Near‐Zero Permittivity Regions Supported by Artificial Organic Hyperbolic Metamaterials

**DOI:** 10.1002/nap2.70235

**Published:** 2026-09-14

**Authors:** José N. Gama, Diogo Cunha, Carla Estévez‐Varela, Marina García‐Pardo, Pablo Pedreira, Adelaide Miranda, M. Carmen López‐González, Pieter A. A. De Beule, Eduardo Solano, Rosalia Serna, Mikhail Vasilevskiy, Martin Lopez‐Garcia, Isabel Pastoriza‐Santos, Sara Núñez‐Sánchez

**Affiliations:** ^1^ CINBIO Universidade de Vigo Campus Universitario As Lagoas Vigo Spain; ^2^ Centro de Física das Universidades do Minho e do Porto (CF‐UM‐UP) Universidade do Minho Campus de Gualtar Braga Portugal; ^3^ International Iberian Nanotechnology Laboratory (INL) Braga Portugal; ^4^ Instituto de Ciencia de Materiales de Madrid (ICMM) Consejo Superior de Investigaciones Científicas (CSIC) Madrid Spain; ^5^ Instituto de Óptica (IO) Consejo Superior de Investigaciones Científicas (CSIC) Madrid Spain; ^6^ ALBA Synchrotron Light Source Cerdanyola del Vallès Barcelona Spain

**Keywords:** anisotropic, exciton polaritons, hyperbolic metamaterial, J‐aggregates, layer‐by‐layer, organic multilayers, surface waves

## Abstract

Hyperbolic metamaterials enable extreme light confinement and control of photonic states, but their realization has been restricted to inorganic architectures. Here, a fully organic route to fabricate artificial hyperbolic metamaterials based on multilayered thin films of J‐aggregate carbocyanine dyes alternated with polyelectrolytes is introduced. These structures exhibit strong optical anisotropy and experimentally support hyperbolic surface exciton polaritons and, for selected dyes, additional surface waves in near‐zero permittivity regimes. Spectroscopic ellipsometry confirms a uniaxial dielectric tensor with negative in‐plane and positive out‐of‐plane components, close to the absorption peaks of the constituent J‐aggregates. This anisotropy is preserved across individual layers, demonstrating the robustness of the layer‐by‐layer approach and enabling the coupling of surface exciton polaritons and surface waves even in films only a few nanometers thick. Transfer‐matrix simulations based on the obtained dielectric tensor reproduce the coupling conditions for all thicknesses, validating the optical model. Structural characterization reveals the link between optical anisotropy and supramolecular order with preferential in‐plane molecular orientation and the evolution from discrete nanostructures to continuous films as deposition progresses. These organic hyperbolic metamaterial architectures, associated with narrow excitonic resonances from J‐aggregates, offer a unique platform for tailoring emission, energy transport, and exploring polariton dynamics at the nanoscale.

## Graphical abstract

A fully organic route to artificial hyperbolic metamaterials is introduced, utilizing multilayered thin films of J‐aggregate dyes. These structures exhibit strong optical anisotropy, experimentally supporting hyperbolic surface exciton polaritons and surface waves in near‐zero Permittivity regions. This architecture provides a robust platform for tailoring emission and energy transport, enabling the exploration of light–matter interactions at the subwavelength regime.

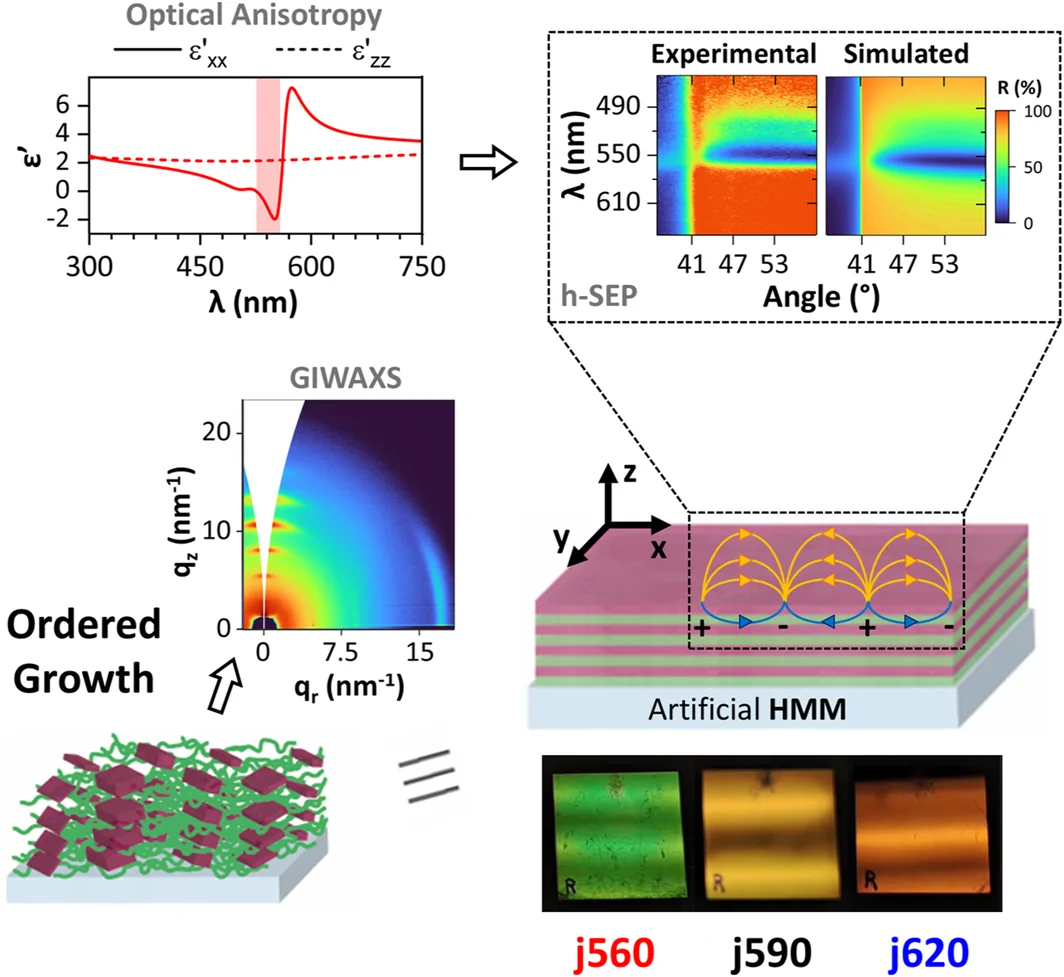

## 1 Introduction

Artificial hyperbolic metamaterials (HMMs) are highly anisotropic media in which the real part of the in‐ and out‐of‐plane components of the dielectric tensor have opposite signs. This unique property leads to a hyperbolic dispersion relation, enabling propagation of high‐wavevector modes and enhanced photonic density of states. Conventional plasmonic metamaterials are typically realized using metal‐dielectric composites, such as multilayers or nanowire arrays, but their tunability is limited by the intrinsic optical properties of the constituent materials [1]. Nevertheless, HMMs are highly attractive for applications ranging from super‐resolution imaging to emission control [2, 3]. However, organic‐based HMMs remain largely unexplored, despite their potential for molecular‐level design and integration into flexible photonic platforms.

Organic semiconductors are a compelling alternative for HMMs, as they often naturally exhibit strong optical anisotropy and can support hyperbolic optical properties. The orientation of the transition dipole momenta within the molecular structure plays a critical role in determining their optical and electronic properties. Hence, the geometrical assembly of the constituent molecules directly impacts the device performance [4, 5]. Several studies have demonstrated that spin‐coated films of polymethine dyes, such as squaraines and quinoidal oligothiophenes, can display natural hyperbolic dispersion due to spontaneous lamellar ordering [6, 7, 8], with negative in‐plane and positive out‐of‐plane components of the dielectric tensor. In these systems, anisotropy arises intrinsically from the spontaneous molecular packing [9]. For example, Barnes and coworkers [10] demonstrated that neat spin‐coated films of a carbocyanine dye—TDBC—exhibit giant bulk optical anisotropy and support hyperbolic surface exciton polaritons (HSEPs), with distinct phase singularities observable via ellipsometric prism coupling.

However, the degree of anisotropy in spin‐coated organic films is highly sensitive to monomer assembly dynamics on the substrate, often limiting control over molecular orientation during deposition [11]. Layer‐by‐layer (LbL) assembly offers a robust alternative for the fabrication of artificial HMMs, enabling precise control over thickness and composition, and is especially suited for chromophores whose assembly can be controlled in solution [12, 13, 14, 15]. J‐aggregates are a notable example; their conformation can be controlled in solution and Frenkel exciton delocalization within each aggregate produces a narrow‐band absorption spectrum [16, 17, 18]. This narrow‐band absorption enables J‐aggregate‐based materials to achieve negative values of the real part of the dielectric tensor [19]. Therefore, combining J‐aggregates with the alternating LbL deposition to construct a true multilayer architecture is especially promising for achieving hyperbolic dispersion, as this approach directly mimics the structural principles used in plasmonic HMMs and enables precise control over the anisotropic optical response.

In this work, we demonstrate the fabrication of fully organic artificial HMMs based on carbocyanine J‐aggregates by optimizing an electrostatic LbL deposition protocol, as a scalable technique extendable to a virtual unlimited number of molecular families. This approach overcomes the limitations of uncontrolled molecular assembly such as spin‐coating [4] in which both in‐plane and out‐of‐plane dielectric tensor components may exhibit strong dispersion depending on the nature of the molecular building blocks [6, 20]. By contrast, the LbL technique promotes uniaxial films with highly anisotropic optical properties by controlling the dipole orientation in each layer. Therefore, by controlling the supramolecular conformation of the dyes in solution, we achieved the deposition of J‐aggregates with a preferential in‐plane orientation of the transient dipole momenta. Our multiscale analysis correlating molecular composition, nanoscale morphology, and molecular ordering confirms that the structural ordering induced by the LbL deposition is responsible for the hyperbolic response in the bulk, which is corroborated by both the anisotropic dielectric tensor obtained by ellipsometry and the excellent agreement between experimental high‐momentum angular reflectivity and transfer‐matrix simulated reflectance. The versatility of the method is demonstrated by obtaining artificial organic HMMs for three different dyes operating at specific wavelengths, with different bulk hyperbolic dielectric tensors, where not only surface exciton polaritons are supported at the interface (named hyperbolic surface exciton polaritons‐HSEP) [10] but also surface waves close to near‐zero permittivity conditions.

## 2 Results and Discussion

### 2.1 Preparation and Characterization of Artificial Organic HMMs

To fabricate artificial organic HMMs, three dyes from a carbocyanine family with J‐aggregate absorption bands at 561, 587, and 619 nm were selected, hereafter referred to as j560, j590 (also known in the scientific community as TDBC), and j620 (Figure 1b). These dyes carry sulfonate groups (Figure 1a), which provide them a net negative electrostatic charge and enable the assembly of J‐aggregate multilayers via the electrostatic LbL method. Using this approach, we alternated the deposition of the negatively charged J‐aggregates with a positively charged polyelectrolyte, such as poly(diallyldimethylammonium chloride) (PDDA), as shown in Figure 1c, to construct highly ordered multilayered thin films on fused silica and borosilicate substrates. Each deposition cycle consisted of immersing the substrate in an aqueous PDDA solution followed by immersion in a J‐aggregate dispersion. This cycle was repeated to achieve precise control over the film thickness, with each cycle referred to as one deposition. J‐aggregate thin films with 1–20 depositions were analyzed individually and a final PDDA layer was deposited to stabilize the last J‐aggregate layer.

**FIGURE 1:**
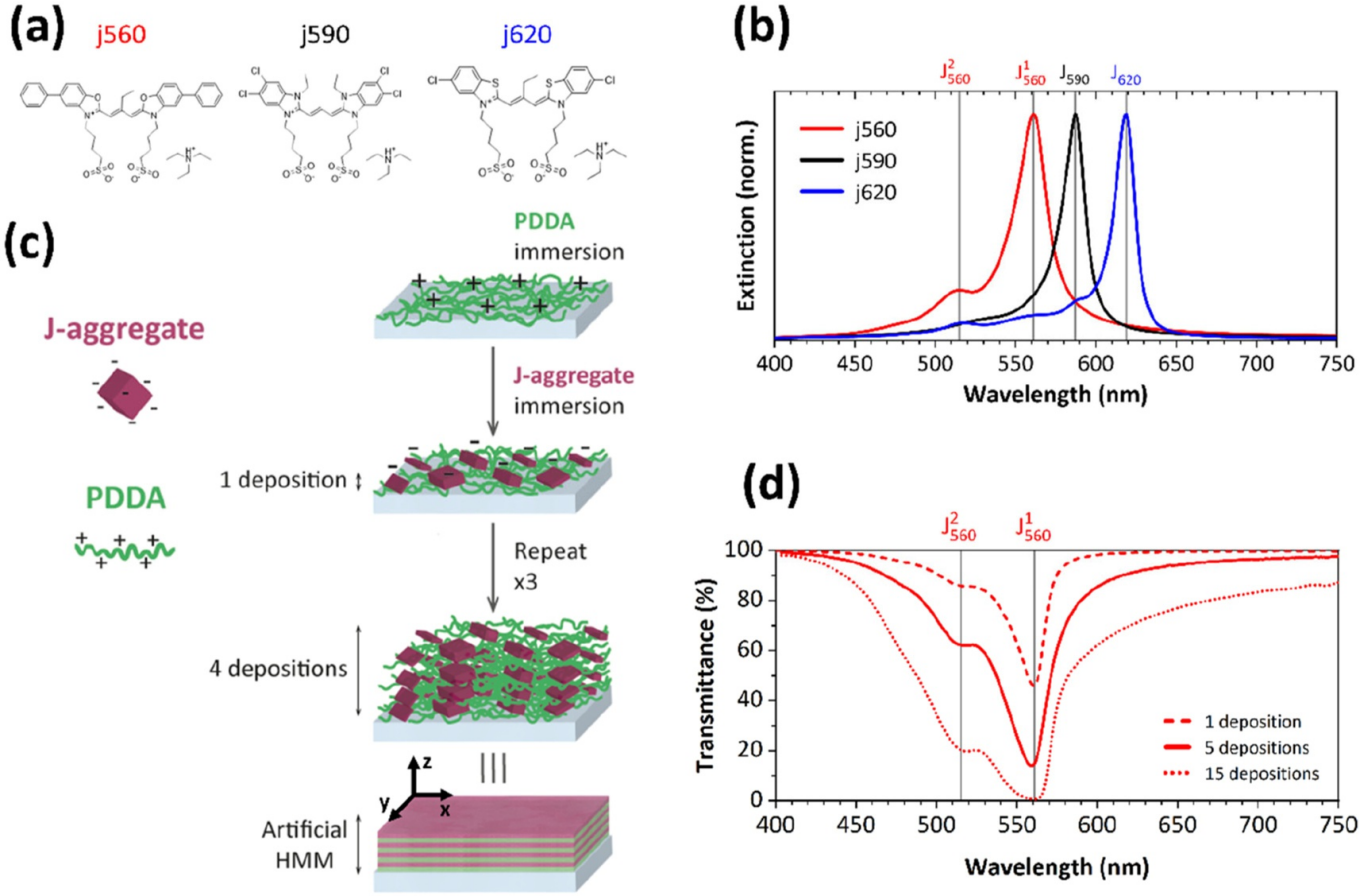
(a) Molecular structure of the selected carbocyanine dyes (j560, j590, and j620). (b) Normalized extinction spectra of the corresponding J‐aggregate dyes in aqueous NaCl solutions. Vertical lines indicate the peak position of the J‐aggregate bands: 515 nm (J^2^_560_) and 561 nm (J^1^_560_) for j560, 587 nm (J_590_) for j590, and 619 nm (J_620_) for j620 (Section 4.3). (c) Scheme of the LbL assembly process for fabricating J‐aggregate thin films (artificial HMMs) illustrating alternating deposition of the positively charged polyelectrolyte (PDDA) and negatively charged J‐aggregates for controlled multilayer growth. (d) Normalized VIS normal incidence transmission spectra of J‐aggregate thin films with different numbers of LbL depositions for j560. Vertical lines match the wavelengths of the peaks associated to the J‐aggregate (indicated as J^1^_560_ and J^2^_560_) conformations of the dyes, targeted in Panel (b).

Transmission of thin films showed a primary minimum at wavelengths corresponding to the J‐aggregate peaks in solution, which is deeper with the number of depositions (Figure 1d and Supporting Information S1: Figure S1). For the case of j560 thin films, two distinct minima at 515 nm (J^1^_560_) and at 561 nm (J^2^_560_) show up, which is consistent with the presence of two J‐aggregate conformations at similar wavelengths that in their spectrum in solution. This analysis confirmed the successful transfer and stabilization of the J‐aggregate in the thin films in each deposition cycle for the three dyes. The increasing broadening of the transmission dip around the dye absorption is related to increased inherent reflectivity (see Supporting Information S1: Figure S2) arising from greater bulk refractive index contrast of the thin film.

The resulting J‐aggregate thin films were analyzed by atomic force microscopy (AFM). Interestingly, AFM topographic 2D images revealed distinct morphological features for the different dyes after the first deposition (Figure 2a). For instance, j560 formed a continuous granular film (Figure 2ai), j590 exhibited grain‐like formations (Figure 2aii), and j620 assembled into ribbon‐like structures (Figure 2aiii). Moreover, the root mean square (RMS) roughness for thin films with 1 deposition cycle increased related to the bare borosilicate substrate, further confirming successful growth of J‐aggregates at the surface after the first deposition (Supporting Information S1: Table S1). For j590 and j620, initial films were discontinuous; however, gaps between structures progressively closed with successive depositions. For example, after 6 depositions, the surface became homogenous, forming a continuous film, where individual supramolecular assemblies were no longer distinguishable (Figure 2bii,iii). AFM analysis also enabled the determination of the film's thickness as a function of the number of depositions. Thin films were scratched in four different areas using a sharp tool and height profiles were measured and averaged. Samples thinner than 3 depositions did not show sufficient continuity to apply a thickness profile tracking, so a quantitative analysis starting from this thickness (Supporting Information S1: Figure S3) revealed near‐linear growth for the three J‐aggregates (Figure 2c). The corresponding growth rates are summarized in Supporting Information S1: Table S1.

**FIGURE 2:**
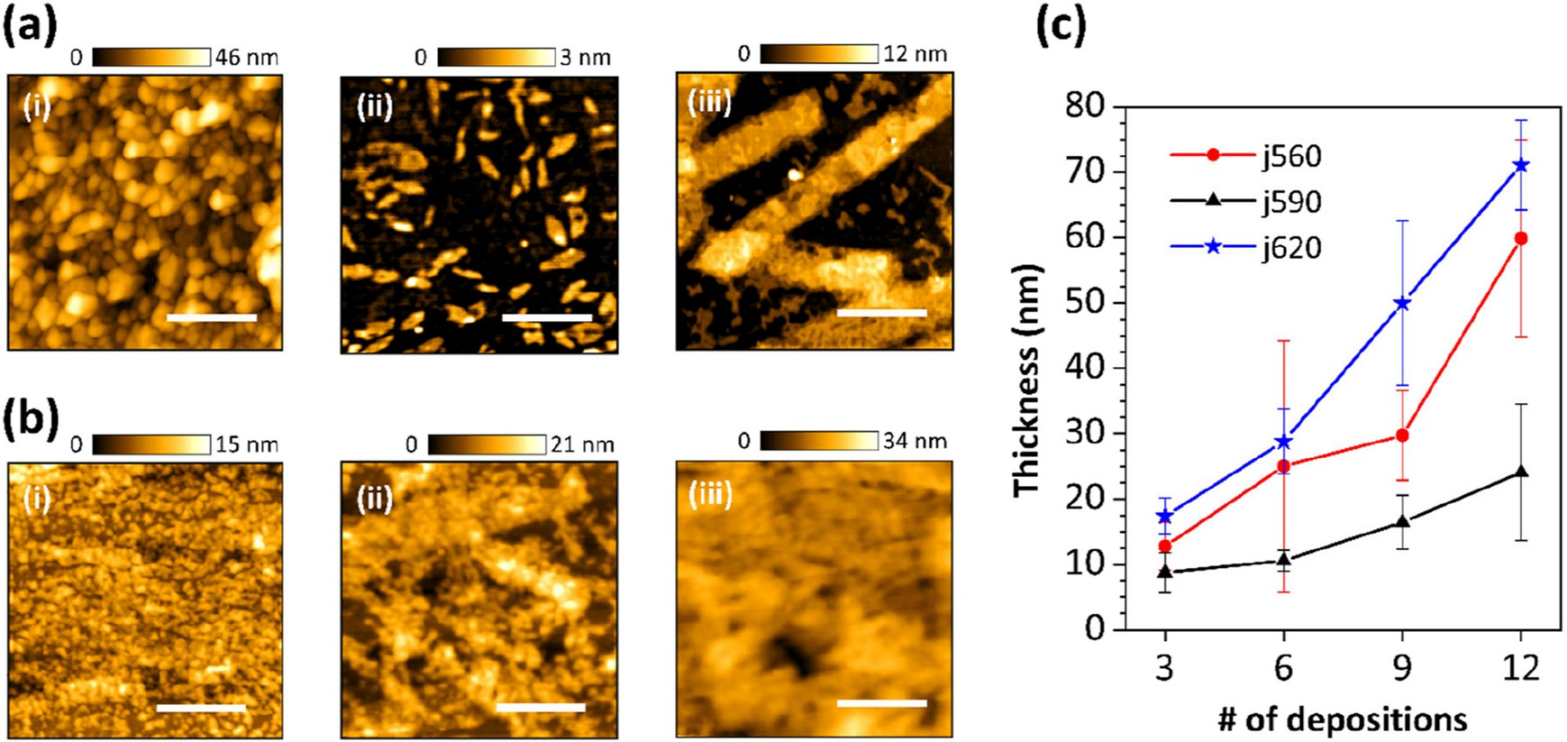
(a–b) AFM topographic 2D images of J‐aggregate thin films after 1 (a) and 6 (b) depositions of j560 (i), j590 (ii), and j620 (iii). Scale bar: 1 μm. (c) Average film thickness as a function of the number of depositions for j560, j590, and j620. Error bars stand for 95% confidence standard deviation in a population of 4 measurements in different sample points.

To gain deeper insights into molecular packing and crystallinity, we performed synchrotron grazing‐incidence wide‐angle X‐ray scattering (GIWAXS) analysis on j560, j590, and j620 thin films with increasing number of depositions (3, 5, 10, and 20). As shown in Figure 3a, the diffraction patterns of j560 display intense discrete peaks, growing in intensity with additional depositions, that is film thickness, primarily along the vertical direction (*q*_*z*_), parallel to the substrate. This growth in intensity is clearer by visualizing the line profiles extracted along vertical and horizontal axes, as shown in Figure 3b. This trend indicates enhanced and coherent lamellar stacking of monomers in the vertical direction. Moreover, the characteristic real space distance (d) was calculated using d=2π/q, where q is the peak position in the reciprocal space. The main *q*_*z*_ peak (Figure 3b, red line) corresponds to a lamellar spacing of 1.30 nm, which is assigned to the internal molecular packing of the ordered J‐aggregate domains. This spacing should not be interpreted as the full LbL structural repeat unit, since AFM measurements show an average j560/PDDA thickness increase of approximately 5 nm per deposition cycle. Additionally, the presence of weaker peaks at 8 and 17 nm^−1^ along the *q*_*r*_ direction (Figure 3b, blue line) is consistent with molecular spacing governed by hydrogen bonding between monomers [21]. Ultimately, the absence of Debye‐Scherrer rings, combined with distinct vertical and horizontal axes in the 2D patterns, provides strong evidence of a highly ordered and anisotropic material. GIWAXS thus suggests a uniaxial optical response, with J‐aggregates randomly oriented in‐plane but exhibiting preferential out‐of‐plane orientation. Consistent diffraction patterns recorded from 3 to 20 depositions confirmed that J‐aggregate orientation is preserved throughout film growth, with intensity increasing with thickness.

**FIGURE 3:**
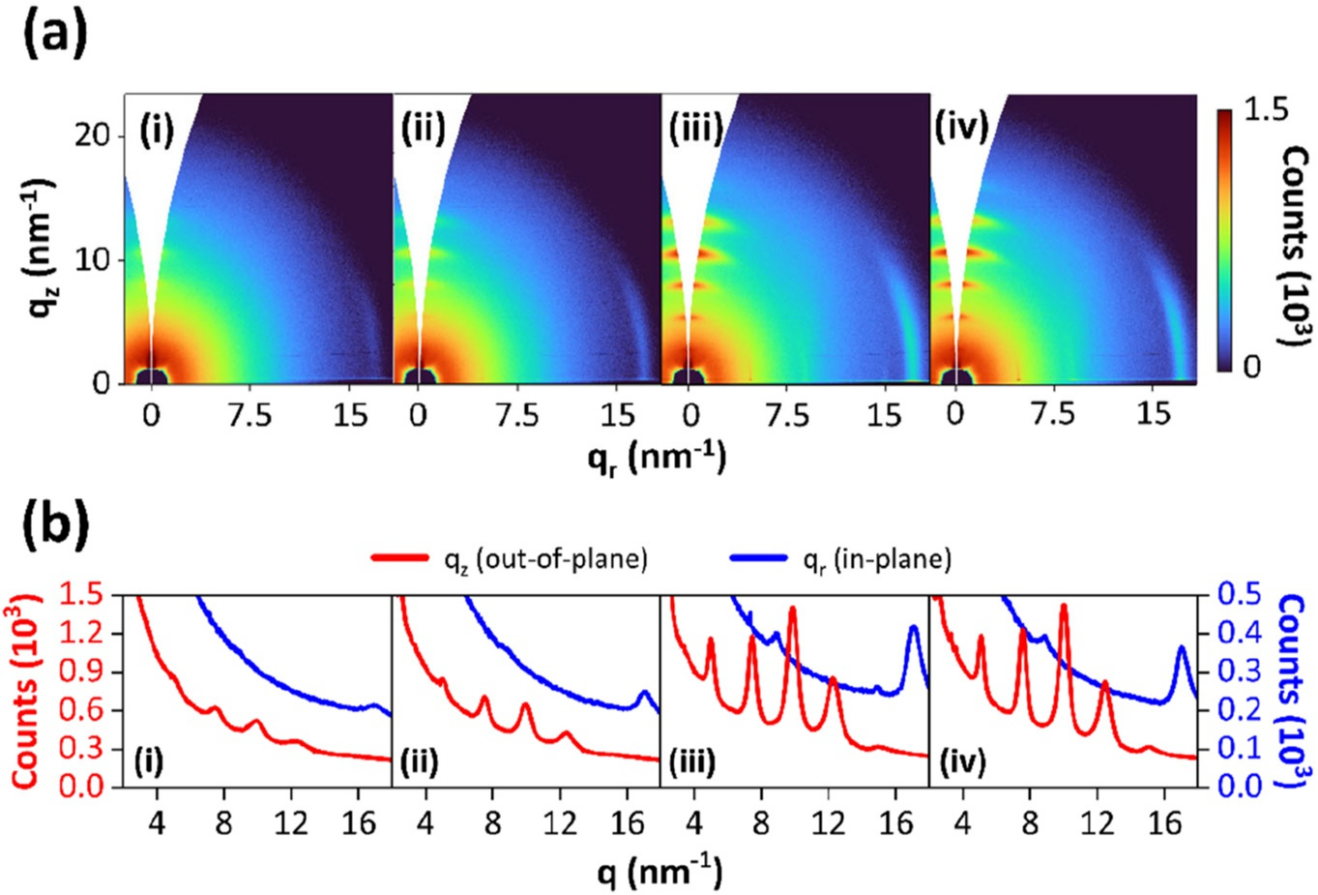
(a) Diffraction patterns obtained by GIWAXS of j560 thin films with (i) 3, (ii) 5, (iii) 10, and (iv) 20 depositions. Horizontal and vertical axes correspond to the wavevector components *q*_*r*_ and *q*_*z*_, respectively. (b) Profiles extracted along the vertical *q*_*z*_ ≈ 0 nm^−1^ (blue) and horizontal *q*_*r*_ ≈ 0 nm^−1^ (red) for the same samples, highlighting the evolution of diffraction peak intensity and position with increasing film thickness.

The same analysis on j590 and j620 films revealed similar trends (Supporting Information S1: Figures S4 and S5). In accordance with AFM measurements of discontinuous early depositions, diffraction peaks appeared only from 3 to 5 depositions, respectively. Both films exhibited the same uniaxial response and progressive intensity increase of diffraction peaks with additional depositions. These results demonstrate the robustness of the LbL deposition technique, as the persistence of identical interference fringes during growth indicates that ordered J‐aggregate domains maintain tight spatial confinement within each layer, ultimately facilitating strong optical anisotropy in the films.

### 2.2 Anisotropic Dielectric Tensor of Artificial Organic HMMs

To further validate the structural anisotropy revealed by GIWAXS, variable‐angle spectroscopic ellipsometry (VASE) was performed on the same set of J‐aggregate thin films. VASE provides quantitative information on the complex dielectric tensors, which are directly influenced by molecular orientation and packing. Measurements were performed on J‐aggregate thin films with 6 and 12 depositions to ensure consistency of the determined complex dielectric tensor (Supporting Information S1: Section S2, Supporting Information, for more details). Thinner samples were not used to extract independent dielectric tensors because of the incomplete surface coverage at the lowest deposition numbers. However, the bulk dielectric tensor components obtained from 12‐deposition films were used to reproduce the ellipsometry parameters, including the transmission response, showing good agreement even for the 1‐deposition samples where the surface is not fully covered. This indicates that the optical response can be described using a common anisotropic dielectric function across thicknesses. However, for the lowest number of depositions, this description should be understood as an effective approximation, as the film is not yet fully continuous. Overall, this confirms the reliability of the LbL deposition method for producing homogeneous bulk properties.

Although J‐aggregates inherently show a specific molecular orientation, our methodology allows for the growth of a multilayered J‐aggregate structure maintaining the same orientation, as stated in Section 2.2, so the dielectric function for the artificial HMMs of the three dyes was modeled as an anisotropic medium with a complex dielectric tensor.

Following the structural analysis of the samples, we consider the artificial organic HMM as a highly uniaxial medium with an in‐plane dielectric tensor $\varepsilon_{xx=yy}$ components and out‐of‐plane dielectric tensor component $\varepsilon_{zz}$ (Supporting Information S1: Figure S6). The real and imaginary parts of the dielectric tensor components were modeled as a combination of Lorentz oscillators, which together reproduce the frequency‐dependent behavior. The model parameters, such as oscillator amplitude, resonance energy, and damping, were optimized for each sample and all tensor components (Supporting Information S1: Table S2), whereas the high‐frequency dielectric constant (ε_∞_) was treated as a direct offset.

Figure 4 shows the in‐plane components of the dielectric tensor with respect to the optical axis of thin films with 12 depositions. As expected for a highly uniaxial material, the in‐plane dielectric tensor $\varepsilon_{xx=yy}$ shows a strong absorption $\varepsilon_{xx}^{\prime \prime }>0$ with a region with $\varepsilon_{xx}^{\prime }<0$, whereas the out‐of‐plane component $\varepsilon_{zz}^{\prime \prime }$ remains nonabsorbing ($\varepsilon_{zz}^{\prime \prime }$ = 0) for the j590 and j620 and only weakly absorbing for j560. This weakly absorbing band may be associated with a small but finite out‐of‐plane transition‐dipole contribution not observed in the GIWAX analysis arising from the coexistence of different J‐aggregate conformations in the j560 films (see Figure 1d). The colored areas in Figure 4a indicate the spectral regions where the dielectric tensor fulfills the hyperbolic condition (${\varepsilon_{xx}}^{\prime }{\varepsilon_{zz}}^{\prime }<\;0$) corresponding to Reststrahlen bands from 526 to 558 nm for j560 (Figure 4ai), 529–583 nm for j590 (Figure 4aii), and 578–613 nm for j620 (Figure 4aiii). Interestingly, for j560, a near‐zero in‐plane permittivity region component (${\varepsilon_{xx}}^{\prime }=0$) with reduced losses separated from the main absorption peak appears between 500 and 528 nm, whereas the out‐of‐plane component remains positive.

**FIGURE 4:**
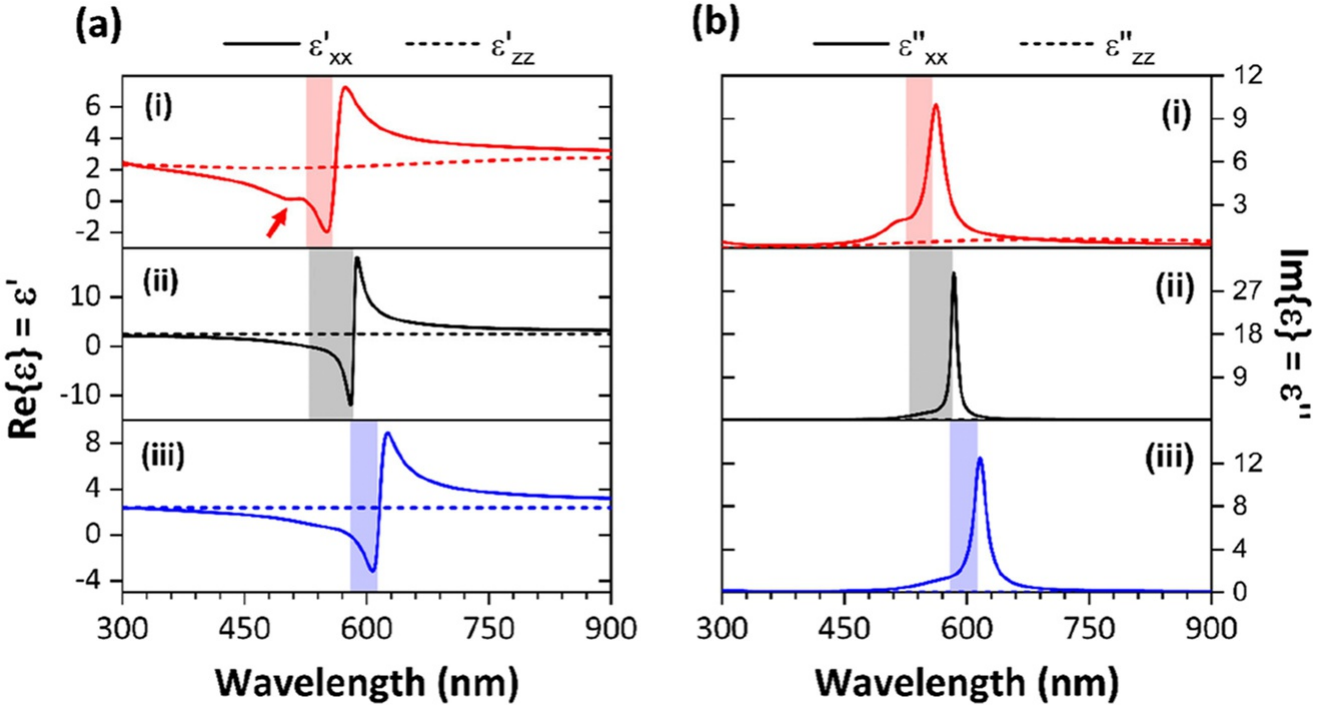
Fitted (a) real *ε*′ and (b) imaginary *ε″* parts of the dielectric tensor for the in‐plane ($\varepsilon_{\text{xx}}$) and out‐of‐plane ($\varepsilon_{\text{zz}}$) components obtained from the ellipsometry analysis of J‐aggregate/PDDA thin films of (i) j560, (ii) j590, and (iii) j620 with 12 depositions. Shaded regions indicate the Reststrahlen bands. The red arrow in (a). (i) indicates the region with near‐zero permittivity values for the case of j560.

### 2.3 Surface Exciton Polaritons and Near‐Zero Permittivity Assisted Surface Waves Supported by Artificial Organic HMMs

In this section, we investigate the surface exciton polaritons supported at the artificial organic HMM/air interface. Following ref. [10], we refer to these interface‐confined modes as HSEPs because they are supported by a medium with a hyperbolic dielectric tensor. Their presence is corroborated by theoretical simulations using the bulk hyperbolic dielectric tensor obtained by ellipsometry in the previous section. Figure 5a shows the reflectance spectra at different collected angles under a Kretschmann prism coupling configuration (see Section 4.9 for more details) for J‐aggregate thin films of the three dyes as a function of the number of depositions [22, 23]. Above the prism critical angle (*θ*_*c*_ = 41.8°), for j590 and j620, distinct single dips in the TM‐polarized reflectance spectra are observed within the Reststrahlen bands of the dielectric's in‐plane tensor. These dips are associated with the coupling conditions of the HSEP mode, where the high‐momentum photons are coupled to the guided mode at the film/air interface and not reflected. The dispersion of these features exhibited a clear dependence on film thickness (number of depositions). For j560 and j620 thin films with 1 deposition, a narrow coupling region is observed, indicating the formation of a well‐defined HSEP mode (Figure 5ai,vii) in a film/nanostructure of just a few nanometers thick. In contrast, j590 required at least two depositions to support any HSEP mode (Figure 5aiv). This difference is attributed to the distinct early stage film morphologies observed by AFM. The first j590 deposition forms sparse grain‐like structures with lower surface coverage, resulting in a weaker effective optical response that is insufficient to produce a detectable HSEP coupling (see Figure 2a). In contrast, j560 forms a more continuous granular film, whereas j620 forms larger ribbon‐like domains, both providing enough local optical contrast to support a weak coupling feature already after one deposition.

**FIGURE 5:**
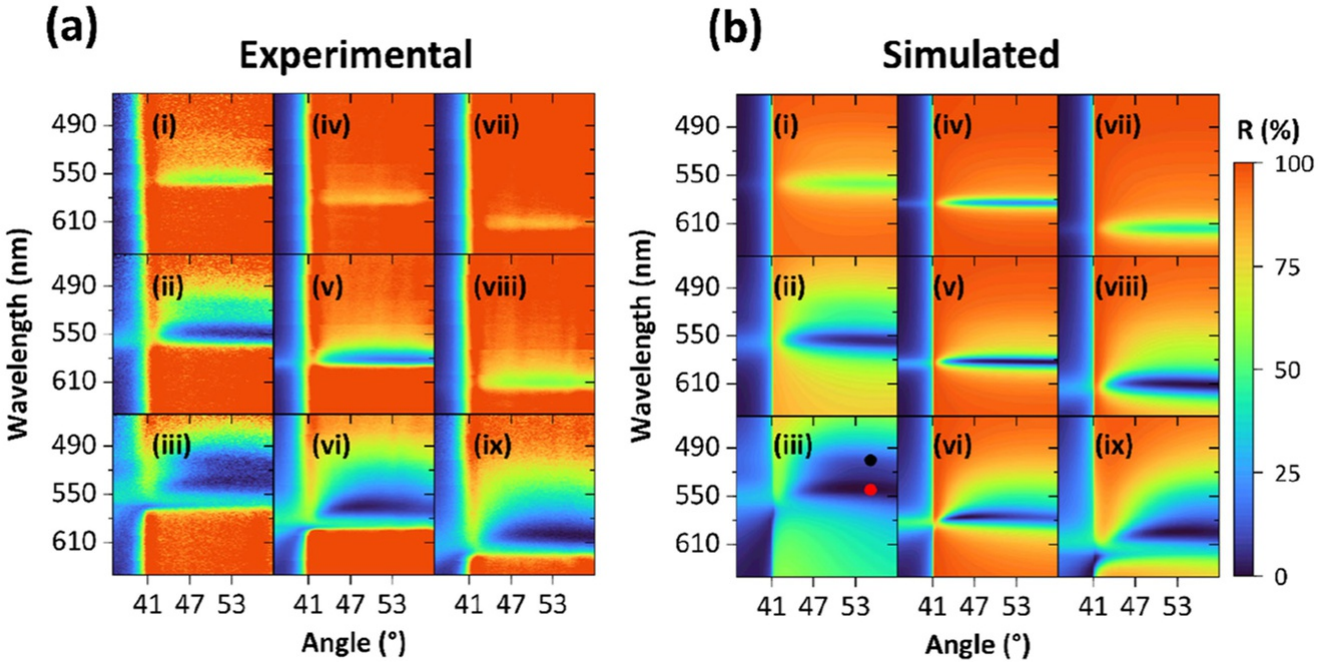
(a) Experimental and (b) simulated TM‐polarized angular reflectance of J‐aggregate thin films under surface wave coupling conditions for increasing deposition cycles. (i) 1, (ii) 5, and (iii) 15 j560 depositions; (iv) 2, (v) 5, (vi) 15 j590 depositions; (vii) 1, (viii) 5, and (ix) 15 j620 depositions. Theoretical modeling was performed using J‐aggregate bulk film models and the anisotropic permittivity obtained by ellipsometry and thicknesses estimated by AFM. Black and red dots were placed at 505 and 540 nm, respectively, corresponding to the reflectance minima obtained for a j560 thin film with 15 depositions at an incident angle of 55°.

As the number of depositions increases, the coupling dips become deeper and broader, and the mode profile evolves into a curved shape. Remarkably, for j560, a second resonance emerges at shorter wavelengths, becoming more pronounced with increasing thickness (Figure 5bii). This second resonance is a distinctive feature only of the j560‐based artificial HMMs. It occurs in the spectral region where the real part of the in‐plane dielectric tensor approaches near‐zero values, whereas the imaginary part remains relatively moderate compared with the main resonant‐loss peak. This finite near‐zero‐$\varepsilon_{\text{xx}}^{\prime }$ window enables an additional NZE‐assisted surface mode at the air/HMM interface. It should be noted that the mere crossing of $\varepsilon_{\text{xx}}^{\prime }$ through zero does not necessarily result in an observable NZE‐assisted surface mode. A finite near‐zero $\varepsilon_{xx}^{\prime }$ spectral window with sufficiently moderate losses is required to allow efficient coupling and avoid overdamping of the mode. For reference, TE‐polarized measurements are shown in Supporting Information S1: Figure S8, revealing only guided mode coupling in TM‐polarized conditions, as is expected for a surface exciton polaritons and surface waves.

To further interpret the experimental data, we simulated the prism coupling conditions with a transfer‐matrix method (TMM) for anisotropic materials using the dielectric tensor obtained by ellipsometry for the films and tabulated borosilicate optical properties for the prism (see Section 4.8 for more details). The film is considered a bulk anisotropic material with a hyperbolic dielectric tensor shown in Figure 4, whereas the thickness was varied according to the AFM‐derived values for each number of depositions. The simulated TM‐polarized reflectance spectra are in excellent agreement with the experimental results for the different deposition numbers (Supporting Information S1: Figure S9 for additional thicknesses), corroborating the consistency of the extracted dielectric function across the investigated thickness range. In both the experiment and simulation, the reflectance dips becoming broader and more pronounced as the number of depositions increases (Figure 5b).

Notably for j560, the characteristic double‐dip feature observed experimentally is well reproduced by the simulations. This demonstrates the robustness of the dielectric tensor of the artificial organic HMMs obtained by ellipsometry, which is consistent and independent of the number of depositions.

Electric field profile distributions were simulated at wavelengths and incidence angles corresponding to minima in TM‐polarized reflectance under prism coupling conditions (Figure 5b). These simulations were performed to determine the nature of the coupled modes supported by the J‐aggregate thin films and the surrounding media. For j590 and j620 films with 10 depositions, the electric field (Supporting Information S1: Figure S10) shows a maximum at the air/film interface and decays exponentially toward both the air and the thin film. This behavior corroborates the presence of a strongly confined surface mode at the interface. The fact that this mode occurs at wavelengths where the real part of the in‐plane dielectric tensor is negative confirms their attribution to HSEPs.

In contrast, j560 thin films exhibited a distinctive double dip feature in the reflectance spectra, indicating coupling to two different modes. For a j560 thin film with 15 depositions (Figure 5biii) at an incidence angle of 55°, the first minimum appears at *λ* = 505 nm (black dot) and the second at *λ* = 540 nm (red dot). At 540 nm, the simulated electric field exhibits a pronounced evanescent decay inside the film and toward the surrounding media (Figure 6a), characteristic of a surface‐bound mode [24]. This dip lies within the Reststrahlen band of the real part of the j560 in‐plane dielectric tensor and can therefore be attributed to the excitation of a HSEP (red dot), as it happens for j590 and j620 films.

**FIGURE 6:**
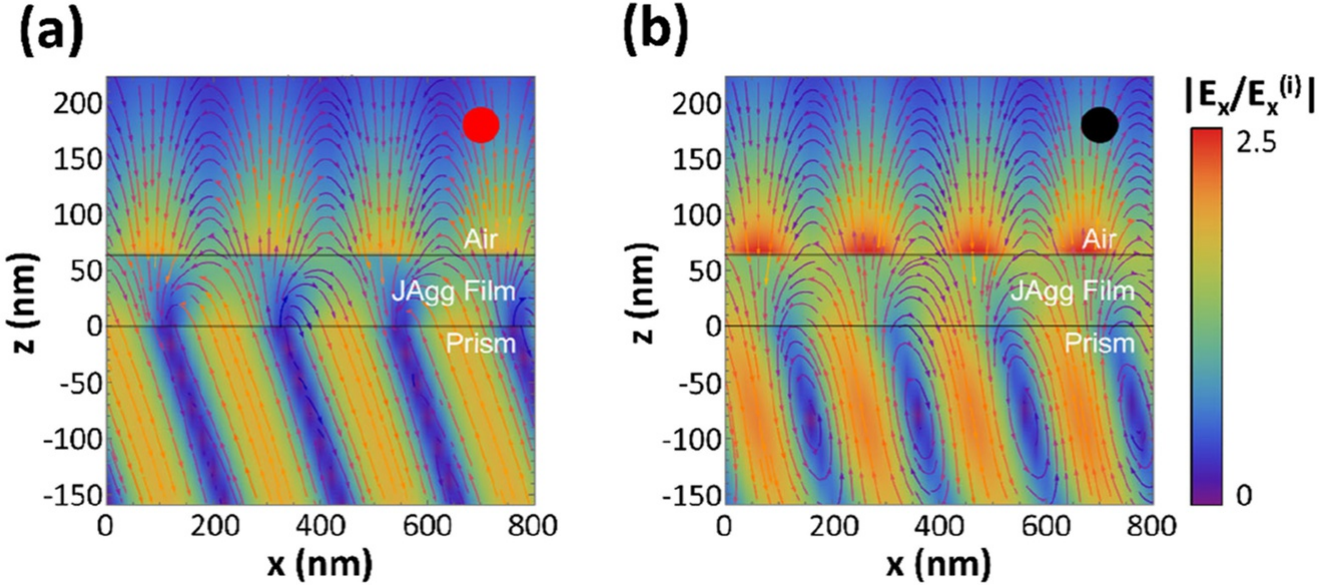
Two‐dimensional colormaps of the modulus of *E*_*x*_ normalized by the incident electric field E^(i)^ in a cross‐section of a j560 thin film with 15 depositions with an incident angle of 55° for (a) *λ* = 540 nm (red line dot in Figure 5biii) and (b) *λ* = 505 nm (black dot in Figure 5biii). Displayed arrows correspond to the electric field vector $\vec{E}$ = (*E*_*x*_,*E*_*z*_).

On the other hand, the first dip at 505 nm corresponds to the near‐zero permittivity region, where the real part of the in‐plane dielectric tensor approaches zero, whereas its imaginary part remains significant, resulting in a partially confined polaritonic mode with extended field penetration (black dot). Thus, the field penetration is more extended, and the evanescent character is less pronounced (Figure 6b). Although the in‐plane dielectric tensor is not negative at this wavelength, it approaches zero while its imaginary part remains significant, leading to a weakly confined, polariton‐like mode supported by the lossy excitonic film. This interpretation is further supported by the *z*‐direction profiles extracted at different *x* values from Figure 6 (Supporting Information S1: Figure S11), and the calculated evanescent wavevector *κ*_2*z*_ inside the film (Supporting Information S1: Table S3), which shows that at 540‐nm the evanescent contribution (real part of the complex momentum) dominates over the propagating one (imaginary part of the complex momentum), whereas at 505 nm, both contributions are comparable. This dual behavior with the coexistence of near‐zero permittivity and hyperbolic regimes in j560 films reveals the versatility of organic artificial HMMs for controlling surface wave modes.

## 3 Conclusions

In this work, we demonstrated a scalable LbL approach to produce artificial organic HMMs based on carbocyanine J‐aggregates and polyelectrolyte linkers, achieving fine control over film thickness, composition, and molecular orientation. Through a comprehensive multiscale analysis, combining AFM, GIWAXS, and VASE, we confirmed the formation of highly ordered, anisotropic thin films with hyperbolic dielectric tensors preserved across individual layers, highlighting the robustness and reproducibility of the method.

Optical measurements revealed that all these HMM thin films support HSEP modes, with their spectral features dependent on the number of LbL depositions. For j590 and j620, the simulated modes are evanescent and well‐confined at the film/air interface, characteristic of HSEPs arising from a negative in‐plane dielectric tensor. In contrast, j560 thin films exhibit a unique double‐dip structure in the reflectance spectra, attributed to the coexistence of two distinct optical regimes: one in the near‐zero permittivity region, supporting a weakly confined polariton‐like mode, and another in the hyperbolic regime, supporting a confined HSEP. Transfer‐matrix simulations based on the experimentally determined permittivity reproduce these coupling conditions across thicknesses, validating the optical model.

Beyond structural and optical validation, these findings establish a scalable route to engineer light–matter interactions and excitonic transport in organic systems, enabling the design and control of distinct surface wave regimes, including hyperbolic exciton polaritons and near‐zero permittivity surface modes. Theoretical simulations based on the transfer‐matrix formalism and ellipsometric parameters show excellent agreement with experimental results. Overall, the strong correspondence between theory and experiment validates the reliability of our approach. Unlike conventional artificial HMMs typically based on inorganic metal‐dielectric architectures, these organic systems synergize hyperbolic dispersion with strong excitonic resonances, offering a unique platform for ultrathin photonic devices, emission control, and exciton–photon coupling in flexible organic architectures.

## 4 Methods

### 4.1 Materials

Poly(diallyldimethylammonium chloride) (PDDA, Mw: 100,000–200,000, 20% wt. in H_2_O) were purchased from Sigma Aldrich. Hydrogen peroxide (H_2_O_2_, 35% wt.) was purchased from Thermo Scientific. Sulfuric acid (H_2_SO_4_, 95% wt.) was purchased from Fisher Scientific. 5‐Phenyl‐2‐[2‐[[5‐phenyl‐3‐(4‐sulfobutyl)‐3H‐benzoxazol‐2‐ylidene]‐methyl]‐but‐1‐enyl]‐3‐(4‐sulfobutyl)‐benzoxazolium hydroxide, inner salt, sodium salt (j560), 5,6‐dichloro‐2‐[[5,6‐dichloro‐1‐ethyl‐3‐(4‐sulfobutyl)‐benzimidazol‐2‐ylidene]‐propenyl]‐1‐ethyl‐3‐(4‐sulfobutyl)‐benzimidazolium hydroxide sodium salt (j590), and 5‐Chloro‐2‐[2‐[5‐chloro‐3‐(4‐sulfobutyl)‐3H‐benzothiazol‐2‐ylidenemethyl]‐but‐1‐enyl]‐3‐(4‐sulfobutyl)‐benzothiazol‐3‐ium hydroxide, inner salt, triethylammonium salt (j620) were provided by FEW Chemicals GmbH. Absolute grade ethanol and Milli‐Q water (18.2 MΩ·cm) were used as solvents. About 1 mm‐thick microscope slides and thickness #1 coverslips were purchased from Marienfeld and Epredia, respectively.

### 4.2 Activation of Silica Substrate Surface

Borosilicate and fused silica substrates were first washed with water and soap to remove grease, then treated with a piranha solution (H_2_SO_4_/H_2_O_2_, 4:1 v/v) for 20 minutes and rinsed twice in Milli‐Q water for 10 minutes each. This treatment eliminates organic contaminants and generates a high density of hydroxyl (‐OH) groups on the surface, increasing surface energy and imparting a uniform negative charge.

### 4.3 Control of Aggregation of Carbocyanine Dyes

The preparation of stable J‐aggregate solutions required careful and systematic optimization of aggregation conditions. For this work, we have selected three carbocyanine dyes, and we labeled them according to the center wavelength of the corresponding J‐aggregates: j560, j590, and j620. For each selected dye, we established a rigorous three‐step optimization protocol to achieve stable J‐aggregate formation. Initial dissolution of the three dyes in ethanol (EtOH) distinct broadband extinction peaks for each (Supporting Information S1: Figure S12). These spectra correspond to a solution of the dyes in the monomer form. Subsequently, when the solvent was changed to water, a dramatic color shift was immediately observed (Supporting Information S1: Figure S13). UV–Vis spectroscopy revealed a red shift of more than 30 nm in the absorption peak compared to the bare monomer (Figure 1b and Supporting Information S1: Figure S11), accompanied by a sharpening of the band, which suggests the formation of ordered J‐aggregate structures. This information was useful to label each dye as monomers according to the center wavelength of the respective J‐aggregate extinction maxima: j560, j590, and j620. Although j590 formed J‐aggregates in Milli‐Q water without additional salts, the optimal aggregation for j560 and j620 required specific ionic strength conditions. A salt concentration screening with Milli‐Q water as solvent identified 50 mM for j560 (Supporting Information S1: Figure S12a) and 100 mM for j620 (Supporting Information S1: Figure S12b) as the ideal concentrations to maximize the J‐aggregate absorption intensity while minimizing contributions from monomers and other colloidal conformations, such as H‐aggregates.

### 4.4 Layer‐by‐Layer Deposition Protocol of Carbocyanine J‐Aggregates

As the activated substrate is negatively charged (see Section 4.2), the first step is its immersion in an aqueous PDDA solution ([PDDA] = 1 mg/mL and [NaCl] = 0.5 M) for at least 15 minutes. After this, the sample is washed thrice in Milli‐Q water (5 seconds in each washing step) and dried with compressed air. The second step is to immerse the sample in a J‐aggregate solution with milli‐Q water as solvent, dye concentration of 300 μM, and an ionic strength of 50, 0, and 100 mM for j560, j590, and j620, respectively (see Section 4.3 for more details). The sample is kept under continuous agitation for 5 minutes to obtain a homogeneous deposition. Afterward, the sample is dried with compressed air, before being washed once with a very quick immersion in an aqueous sodium chloride solution with the same concentration as in the optimized solutions for J‐aggregation of each dye. Next, the sample is immersed with a very quick dip in the aqueous PDDA solution ([PDDA] = 1 mg/mL and [NaCl] = 0.5 M). At this point, we reach 1 deposition, and this cycle can be repeated n‐times to achieve a thin film of *n*+1 depositions of J‐aggregate/PDDA. J‐aggregate solutions must be prepared fresh from 5 to 5 depositions to ensure precise concentration of the dye.

### 4.5 Thin Film Characterization

UV–Vis absorption spectra were obtained using an Agilent 8453 UV–Vis spectrophotometer with glass and quartz cuvettes of 1 mm optical path. Transmission spectra were obtained using an Agilent Cary 5000. Atomic force microscopy (JPK Nanowizard 3 AFM, Bruker Nano GmbH, Germany) topographic measurements in quantitative imaging mode were obtained using a standard tapping mode PPP‐NCHR (NanoSensors) out‐of‐the‐box cantilevers. The cantilevers with a nominal spring constant of 42 N/m have an aluminum coating layer on the detector side and a nominal frequency of 330 kHz. AFM images were acquired in quantitative imaging mode using a setpoint of 100–200 nN and a z‐length of 60–300 nm. Image analysis was performed using Gwyddion 2.67.

### 4.6 Grazing‐Incidence Wide‐Angle X‐Ray Scattering

GIWAXS measurements were performed at the ALBA synchrotron to investigate the crystallographic crystalline structure and molecular ordering of the J‐aggregate thin films. The technique was selected for its well‐known sensitivity to surface and near‐surface structural features, which is particularly advantageous for characterizing very thin samples. For that, an X‐ray beam of 12.4 keV was produced by an in vacuo undulator with 21 mm period and 93 periods, and it was further monochromatized using a Si (111) channel cut monochromator. An array of Be compound refractive lenses (CRLs) was employed to collimate the beam, obtaining a beam size of 50 × 150 μm^2^ (*V* × *H*) FWHM at the sample position. A stack of motors ensured precise tilts and translation of the sample in the beam. A Rayonix LX255‐HS detector with a pixel array of 2880 × 960 (*V* × *H*) pixels with a size of 88.54 × 88.54 μm^2^ was employed to record the scattering patterns. The sample to detector distance and detector tilts were calibrated using Cr_2_O_3_ from NIST as a calibration standard, allowing to generate the reciprocal space maps. To optimize the detection of diffraction peaks, especially in films with low deposition density, we systematically varied the grazing angle of incidence during the measurements. Only the optimized datasets, corresponding to angles that maximized the signal‐to‐noise ratio and peak visibility, are presented in this work: Figure 3, (i) 0.13°, (ii) 0.13°, (iii) 0.13°, and (iv) 0.11°; Supporting Information S1: Figure S4 (i) 0.13°, (ii) 0.15°, (iii) 0.12°, and 0.13° (iv); and for Supporting Information S1: Figure S5 (i) 0.13°, (ii) 0.13°, and (iii) 0.11°.

### 4.7 Variable‐Angle Spectroscopic Ellipsometry

Spectroscopic ellipsometry characterization was performed on the J‐aggregate‐PDDA thin films deposited on fused silica substrates, in the 250–1700 nm wavelength range using a J. A. Woollam VASE system. The measurements of the ellipsometric parameters, *Y* and *D*, were done at three angles of incidence of 65°, 70°, and 75°. Additionally, transmission (*T*) spectra at normal incidence (0°) were performed in the same film structure. We performed simultaneous fitting of the *Y*, *D,* and *T* spectra with the transfer matrix formalism considering the films as an effective, homogeneous film on a semi‐infinite fused‐silica substrate. The dielectric tensor was modeled as uniaxial anisotropic to determine the in‐plane ($\varepsilon_{∥}$) and out‐of‐plane ($\varepsilon_{⊥}$) components. The in‐plane dielectric tensor ($\varepsilon_{∥}$) for each j‐aggregate type was built as a sum of Lorentzian oscillators to ensure Kramers–Kronig consistent analysis. For the out‐of‐plane ($\varepsilon_{⊥}$) component for the films prepared with the j590 and j620 j‐aggregates, it has found that $\varepsilon_{⊥}$ = constant in all the studied range, whereas for the film prepared with j560, we found that it showed a weak and broad absorption that was well modeled with a Gaussian oscillator (Supporting Information S1: Section S2). The best fit to the measured values was achieved with a Levenberg–Marquardt algorithm using the WVASE software. This has been proven to be a successful approach to determine the complex dielectric tensor when many fitting parameters are involved [25].

### 4.8 Attenuated Total Reflectance and Electric Field Profile Calculations

The numerical calculations of the attenuated total reflection (ATR) spectra and electric field profiles shown were performed under TMM written in‐house. Both reflectance maps and field profiles were derived from analytical equations described in Supporting Information S1: Section S3 of the Supporting Information, where more detailed information about the used models is written.

### 4.9 Surface Waves Coupling Measurements

The angle and polarization resolved reflectance of the J‐aggregate LbL films was obtained using the Fourier imaging spectroscopy (FIS) technique. A halogen lamp covering the UV–Vis–NIR spectral range was focused down to a controllable spot size (∼20 μm) by a high NA lens (Nikon 100 × Plan Apo NA = 1.45) that allowed the selection of single areas over the sample. The reflectance of the thin films was then collected by the same high NA lens in an epi‐illumination configuration, and the back focal plane image of the lens was projected into a UV–Vis spectrograph (Princeton Instruments, Acton SpectraPro SP‐2150 with attached CCD camera QImaging Retiga R6 USB3.0 Color). HSEP propagation at the interface can only be launch by matching the parallel momentum of the incident beam to that of the surface wave characteristic of an evanescent electric field at the J‐aggregate/air interface. The high NA oil immersion lens (Nikon 100 × Plan Apo NA = 1.45) allows both incidence and collection of reflected light with angles between −72.78° and 72.78°. Under this configuration, light was coupled to electromagnetic excitation in the thin film surface under Kretschmann prism‐coupling equivalent configuration, which ensures coupling with wavevectors beyond the glass/air light line in k‐space. The collected spectra were normalized by the spectra of a silver‐coated mirror model ‐P01 > 97% from THORLABS Inc. measured under the same conditions as the sample. The final normalized data were corrected by the reflectance spectra of the mirror calibrated by the producer at an angle of incidence (AOI) of 45°. The films were systematically prepared with increasing numbers of depositions on #1‐thickness borosilicate coverslips (∼0.17 mm‐thick) for surface wave coupling measurements, considering the small working distance of the objective lens.

## Author Contributions

**José N. Gama:** writing – original draft, investigation, validation, formal analysis, data curation. **Diogo Cunha:** investigation, writing – original draft, validation, formal analysis. **Carla Estévez‐Varela:** investigation, formal analysis. **Marina García‐Pardo:** investigation, data curation, formal analysis. **Pablo Pedreira:** investigation, validation, formal analysis, data curation. **Adelaide Miranda:** investigation, validation, formal analysis, data curation. **M. Carmen López‐González:** investigation, validation. **Pieter A. A. De Beule:** investigation, validation, formal analysis, resources. **Eduardo Solano:** investigation, conceptualization, methodology, validation, formal analysis, data curation. **Rosalia Serna:** conceptualization, investigation, validation, funding acquisition, writing – review and editing, methodology, supervision, resources. **Mikhail Vasilevskiy:** funding acquisition, investigation, conceptualization, writing – review and editing, validation, methodology, supervision, **Martin Lopez‐Garcia:** conceptualization, investigation, funding acquisition, methodology, validation, visualization, writing – review and editing, supervision, resources. **Isabel Pastoriza‐Santos:** conceptualization, investigation, funding acquisition, methodology, validation, visualization, writing – review and editing, supervision, resources. **Sara Núñez‐Sánchez:** investigation, conceptualization, funding acquisition, writing – original draft, writing – review and editing, validation, methodology, visualization, supervision, resources.

## Supporting information


Supporting Information S1

## Data Availability

The data that support the findings of this study are openly available in Zenodo at https://doi.org/10.5281/zenodo.21532894.
