# Supplementary material for: Hyperbolic Surface Exciton Polaritons and Surface Waves in Near‐Zero Permittivity Regions Supported by Artificial Organic Hyperbolic Metamaterials
Source: Nanophotonics. 2026 Sep 14;15(18):e70235. doi: 10.1002/nap2.70235 (PMC13672131; doi:10.1002/nap2.70235)
Supplement: Supplementary file 1 [file NAP2-15-e70235-s001.pdf]

Supporting Information

Hyperbolic Surface Exciton Polaritons and Surface Waves in Near-Zero Permittivity Regions Supported by Artificial Organic Hyperbolic Metamaterials

José N. Gama, Diogo Cunha, Carla Estévez-Varela, Marina García-Pardo, Pablo Pedreira, Adelaide Miranda, M. Carmen López-González, Pieter A.A. De Beule, Eduardo Solano, Rosalia Serna, Mikhail Vasilevskiy, Martin Lopez-Garcia, Isabel Pastoriza-Santos, Sara Núñez-Sánchez**

Section S1. Preparation and Structural Analysis of Artificial Organic HMMs

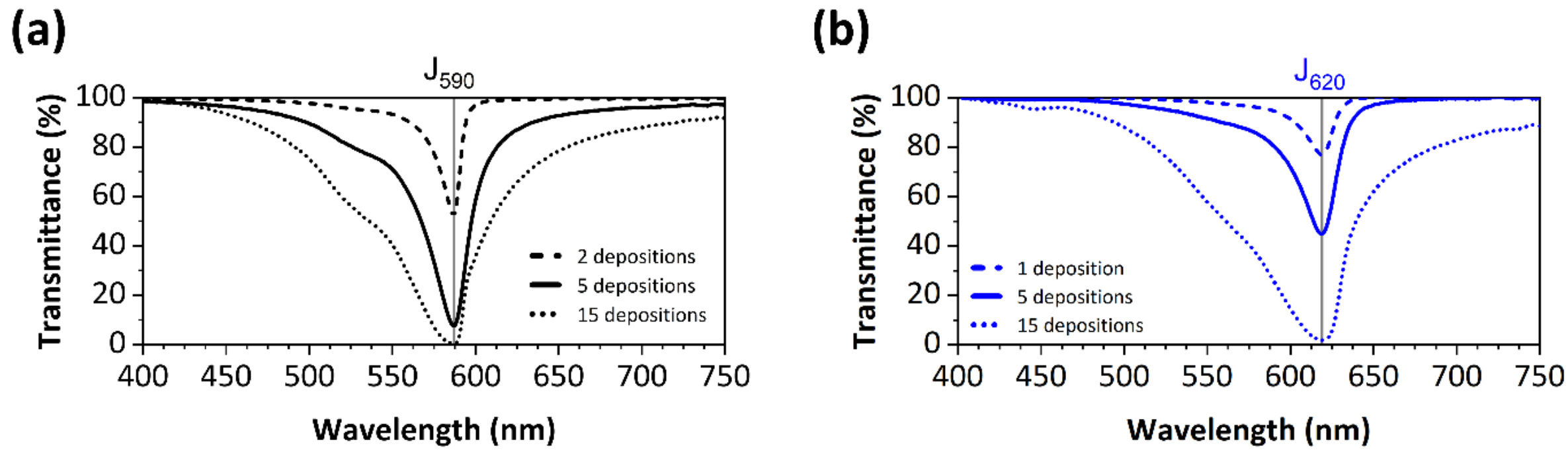

Figure S1. Normalized VIS normal incidence transmission spectra of J-aggregate thin films with different number of deposition cycles for: **(a)** j590 and **(b)** j620. Vertical lines match the wavelengths of the peaks associated to the J-aggregate (indicated as J_{590} and J_{620}) conformation of the dyes, targeted in Figure 1b.

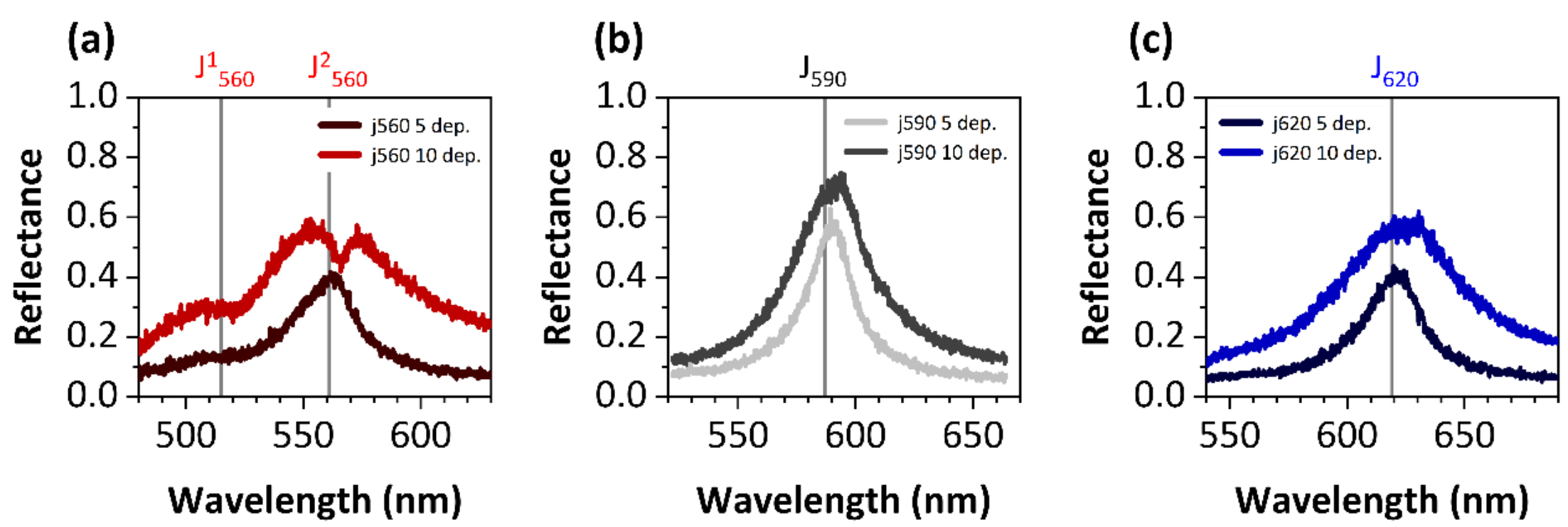

Figure S2. Quasi-normal incidence ($\theta_{incidence} \approx 3°$) reflectance J-aggregate thin films with different number of deposition cycles for: **(a)** j560 and **(b)** j590, and **(c)** j620. Vertical lines match the wavelengths of the peaks associated to the J-aggregate (indicated as J^1_{560}, J^2_{560}, J_{590}, and J_{620}) conformation of the dyes, targeted in Figure 1b.

Table S1. Comparison of statistical data of thickness per deposition and RMS roughness of the first LbL deposition, obtained by analysis of AFM topographic 2D images of the thin films.

	**Borosilicate**	**j560**	**j590**	**j620**
**Thickness** /dep. (nm)	**-**	**4.25** ± 1.49	**2.00** ± 0.64	**5.58** ± 0.91
RMS roughness $\mathbf{r_q}$ (nm)	**0.35** ± 0.06	**7.25** ± 0.69	**0.46** ± 0.18	**2.58** ± 1.29

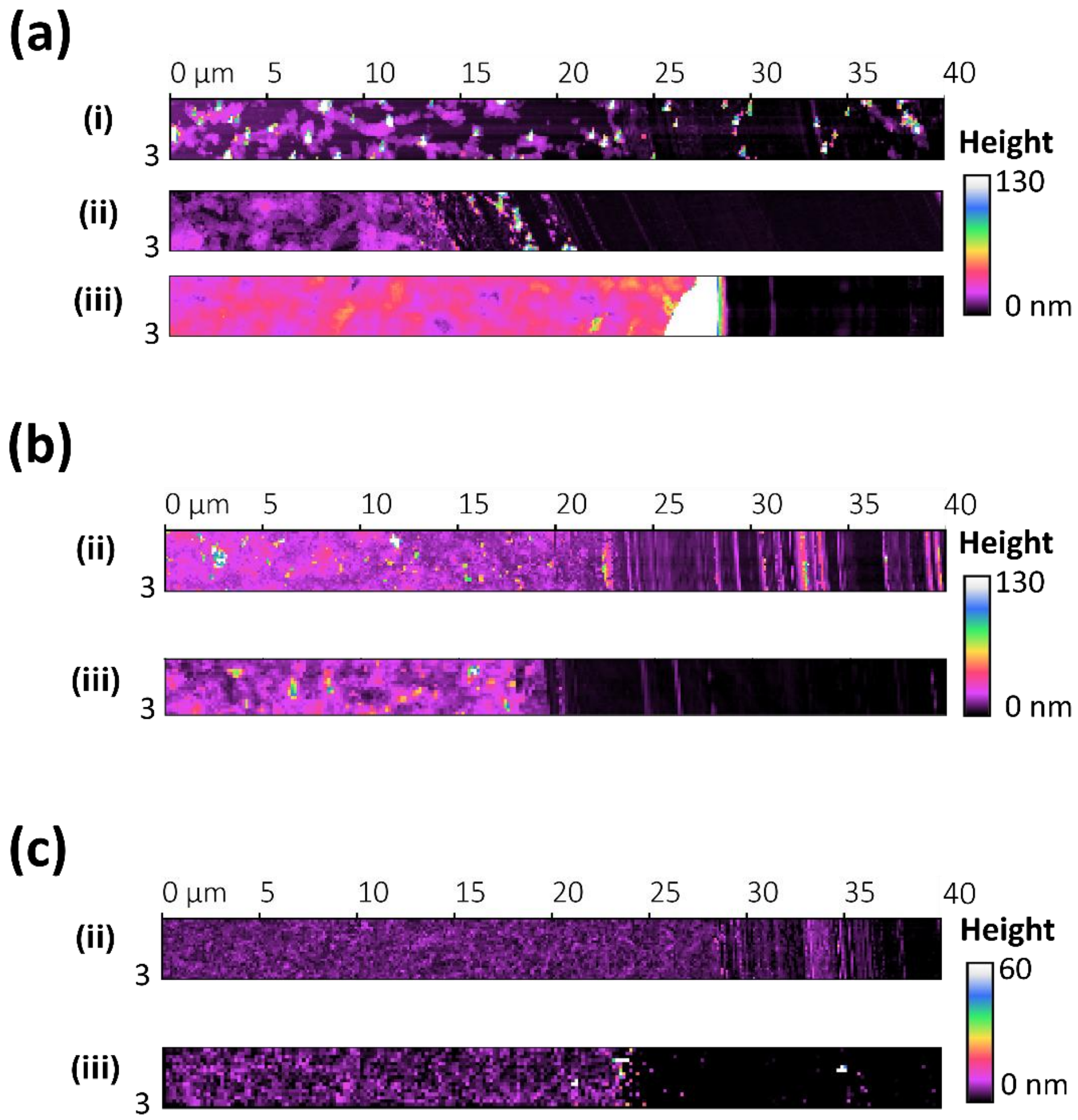

Figure S3. AFM height maps over scratched surface on thin films of **(a)** j620, **(b)** j560, and **(c)** j590 with: (i) 1, (ii) 3, and (iii) 6 depositions.

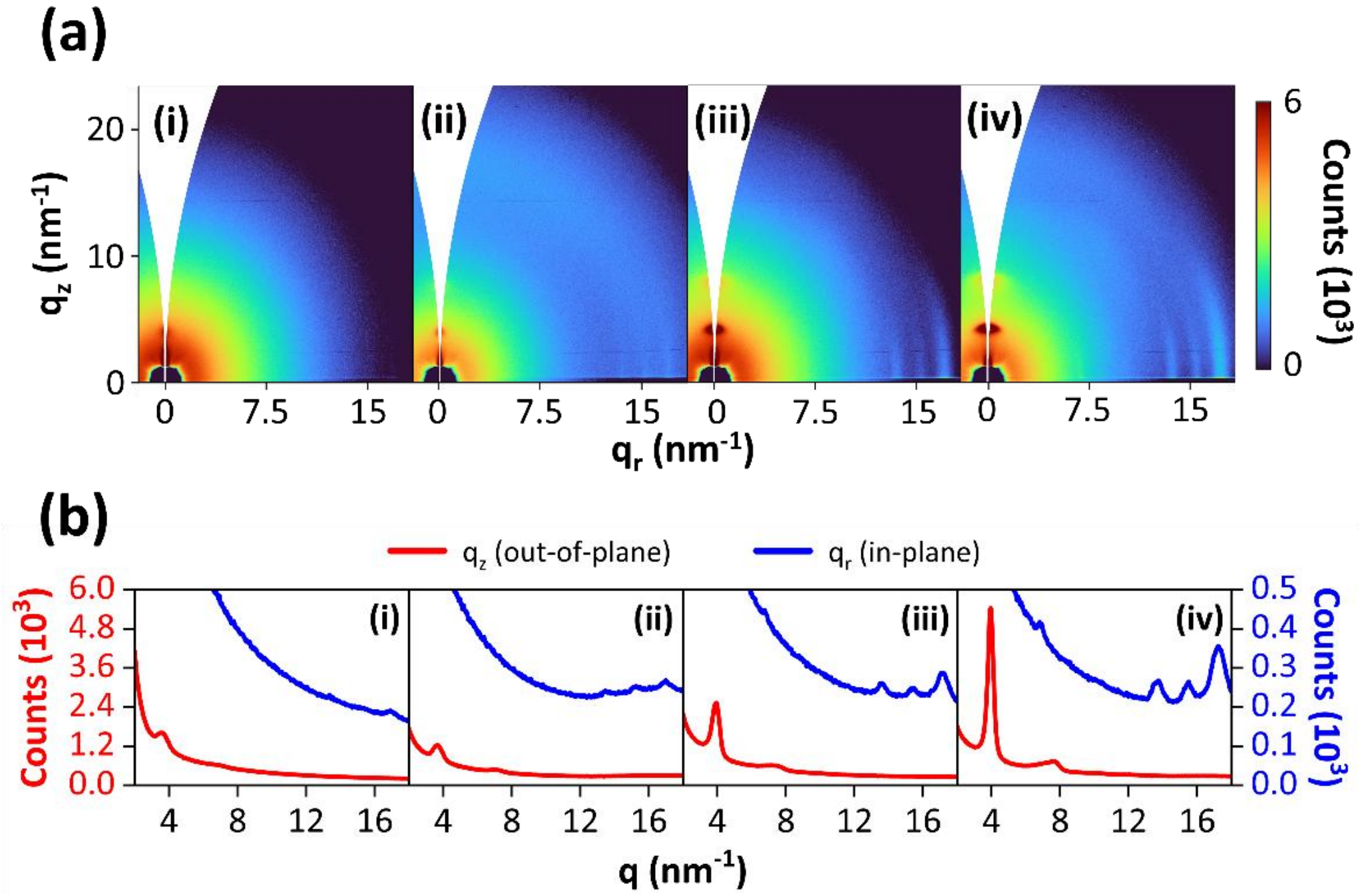

Figure S4. (a) Diffraction patterns obtained by GIWAXS of LbL thin films of j590 with (i) 3, (ii) 5, (iii) 10, and (iv) 20 depositions. Vertical and horizontal axes correspond to the wavevector components q_r and q_z, respectively. **(b)** Profiles taken from Figure 3a at near horizontal $q_z \approx 0$ nm^{-1} (blue) and vertical $q_r \approx 0$ nm^{-1} (red) for (i) 3, (ii) 5, (iii) 10, and (iv) 20 depositions. The presence of a mild halo at $q_r = 16$ nm^{-1} in figure S3a.(ii) is due to contributions of the substrate as the angle of incidence was tilted by 0.02° for better visualisation of the diffraction peaks.

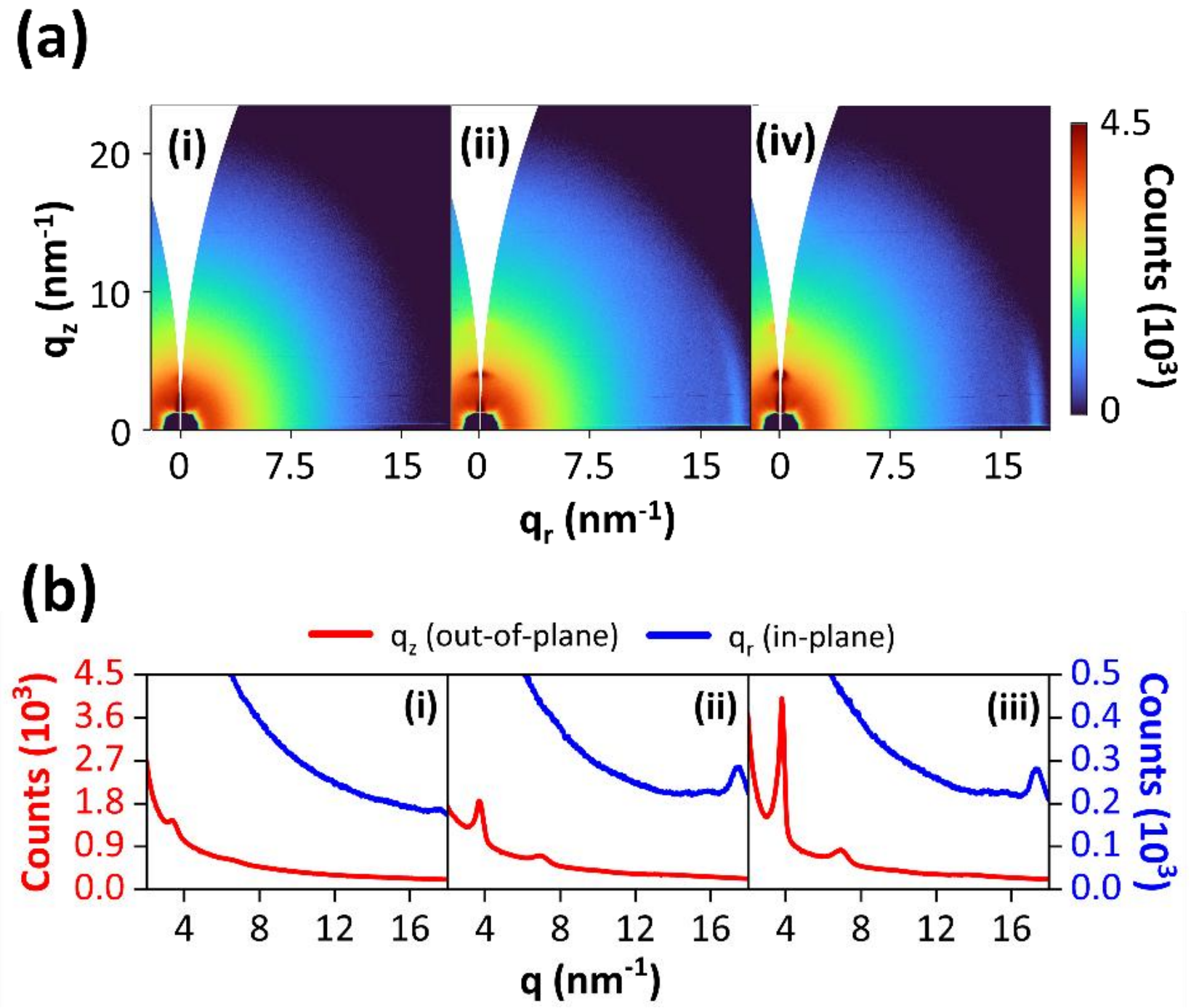

Figure S5. (a) Diffraction patterns obtained by GIWAXS of LbL thin films of j620 with (i) 5, (ii) 10, and (iii) 20 depositions. Vertical and horizontal axes correspond to the wavevector components q_r and q_z, respectively. **(b)** Profiles taken from Figure 3a at near horizontal $q_z \approx 0$ nm^{-1} (blue) and vertical $q_r \approx 0$ nm^{-1} (red) for (i) 5, (ii) 10, and (iii) 20 depositions.

Section S2. Optical response of Artificial Organic HMMs

Ellipsometry analysis

The determination of the dielectric function of the J-aggregate/PDDA thin films was carried out by the analysis of VASE measurements. The ellipsometry measurements allow to obtain the Ψ and Δ angles that are defined as:

$$\tan(\Psi)\, e^{i\Delta} = \frac{\tilde{r}_p}{\tilde{r}_s}$$

where $\tilde{r}_p$ and $\tilde{r}_s$ are the Fresnel coefficients for the parallel and perpedicular components respect to the plane of incidence. With the ellipsometer we measure $\tilde{r}_p$ and $\tilde{r}_s$ as a function of wavelength and angle of incidence (θ).

Accurate fittings of the measured Ψ and Δ values for the multilayered J-aggregate/PDDA thin film structures were obtained using the transfer matrix formalism and modelling the films with uniaxial anisotropy. In this model, the films are treated as uniaxial layers with the optical axis perpendicular to the substrate, as shown in Figure S5.

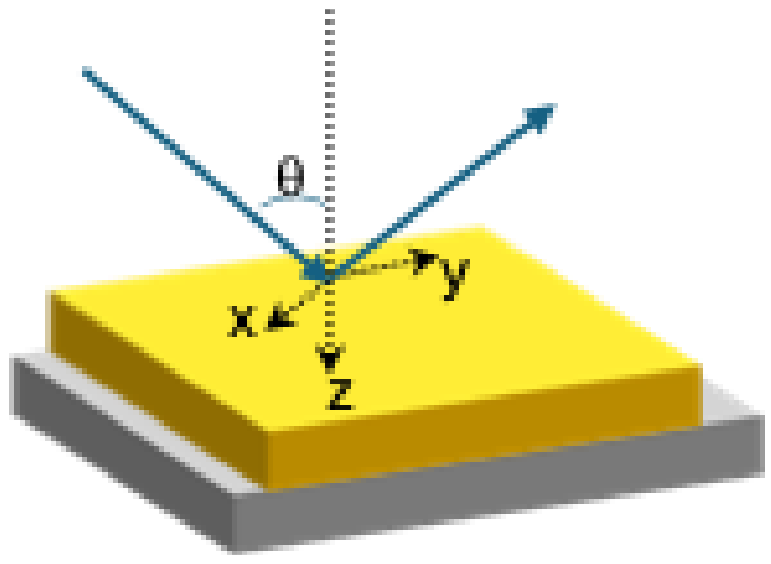

Figure S6. Scheme showing the axis defined for the uniaxial J-aggregate/PDDA thin film configuration. The optical axis is parallel to the z axis. The x and y axis define the film plane. The blue arrows show the incident light at an angle θ and define the plane of incidence.

According to this configuration, we define the in-plane dielectric tensor as $\varepsilon_{xx=yy}$ and the out-of-plane dielectric tensor as ε_{zz}. The corresponding dielectric tensor is therefore:

$$\boldsymbol{\varepsilon} = \begin{pmatrix} \varepsilon_{xx} & 0 & 0 \\ 0 & \varepsilon_{xx} & 0 \\ 0 & 0 & \varepsilon_{zz} \end{pmatrix}$$

To obtain the dielectric function for the J-aggregate/PDDA thin films of the three studied J-aggregate derivatives: j560, j590, and j620; we used a combination of oscillators which together reproduce the frequency-dependent behaviour of the complex dielectric function:

$$\varepsilon_n = \varepsilon_n' + i\varepsilon_n''$$

Each oscillator term contributes to the overall dielectric response and is expressed, in the Lorentzian form, as:

$$\varepsilon_n(\omega) = \frac{f_n \gamma_n \omega_n}{\omega_n^2 - \omega^2 - i\gamma_n \omega}$$

and in the Gaussian form, as:

$$\varepsilon_n(\omega) = f_n \exp\left[-\frac{(\omega - \omega_n)^2}{2\gamma_n^2}\right]$$

where f_n is the oscillator strength, ω_n the resonance frequency, and γ_n the damping constant.

The final dielectric function is therefore obtained as a sum of the Lorentzian and Gaussian oscillators:

$$\varepsilon\,(\omega) = \varepsilon_\infty + \sum_n \varepsilon_n(\omega)$$

This approach ensured the full Kramers-Kronig consistency. Exceptionally, for example for the out-of-plane dielectric tensor of the j560 derivative, the experimental spectra exhibited broader absorbing features that were better described by a Gaussian oscillator. The fitting parameters and the corresponding oscillator types used for each film are listed in Table S10.

Table S2. Fitted parameters for the in-plane ($\varepsilon_{xx=yy}$) and out-of-plane (ε_{zz}) dielectric tensor components of j560, j590, and j620 thin films with 12 depositions.

j560					j590					j620				
In-plane component of dielectric tensor ($\varepsilon_{xx=yy}$)														
**Oscillator**	**f_n**	**ω_n (eV)**	**γ_n (eV)**	**ε_∞**	**Oscillator**	**f_n**	**ω_n (eV)**	**γ_n (eV)**	**ε_∞**	**Oscillator**	**f_n**	**ω_n (eV)**	**γ_n (eV)**	**ε_∞**
**Lorentz**	9.9	2.21	0.09		Lorentz	28.9	2.12	0.03		Lorentz	12.0	2.01	0.06	
**Lorentz**	1.0	2.41	0.22	1.82	Lorentz	1.2	2.24	0.28	1.88	Lorentz	8.0	2.18	0.35	2.14
**Lorentz**	5.0	2.08	0.10		Gauss	9.1	6.33	0.46		Lorentz	0.9	4.03	0.55	
**Lorentz**	2.3	4.83	0.63		-	-	-	-	-	Gauss	0.9	6.14	2.15	
Out-of-plane component of dielectric tensor (ε_{zz})														
**Oscillator**	**f_n**	**ω_n (eV)**	**γ_n (eV)**	**ε_∞**	**Oscillator**	**f_n**	**ω_n (eV)**	**γ_n (eV)**	**ε_∞**	**Oscillator**	**f_n**	**ω_n (eV)**	**γ_n (eV)**	**ε_∞**
**Gauss**	0.6	1.75	1.42	2.41	No Oscillator	-	-	-	2.50	No Oscillator	-	-	-	2.42

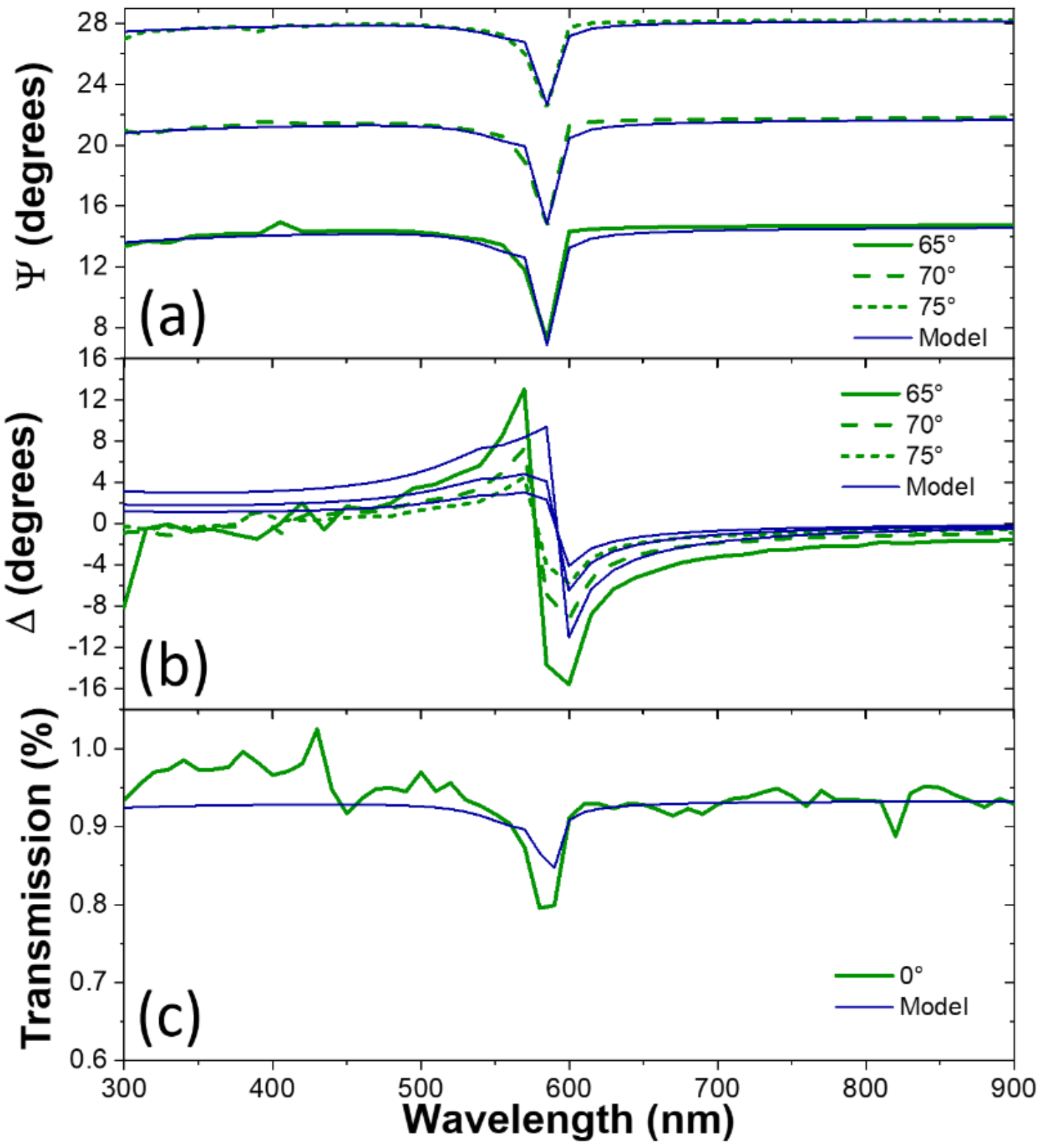

Figure S7. Measured "Ψ" **(a)** and "Δ" **(b)** values at 65°, 70°, and 75° incidence angle (green lines) and measured transmission at normal incidence **(c)** (green line) compared with the fitting data obtained using the dielectric tensor obtained for the film of 12 depositions (blue lines).

Section S3. Transfer Matrix formalism for anisotropic media

To compute the attenuated total reflectance (ATR) spectra for a setup closely resembling the experimental configuration in a consistent approach with the previous section, which is described in detail on section 4.9 of the main manuscript, we have modelled the J-aggregate/PDDA films as a uniaxial anisotropic layer, characterized by the dielectric tensor

shown in section S2, where $\varepsilon_{xx=yy}$ and ε_{zz} are the in-plane and out-of-plane dielectric tensors obtained from the ellipsometry analysis, respectively.

The scheme consists of a layer of oil serving as the prism, followed by a refractive index-matching glass coverslip. Beyond the coverslip lies the uniaxial J-aggregate film, and finally, a semi-infinite air medium. In this arrangement, the evanescent field generated at the prism interface couples to the excitonic transitions within the J-aggregate/PDDA thin film, leading to the formation of SEPs localized at the film-air interface. Since the refractive index of the immersion oil is chosen to match that of the glass substrate, this setup can be classified as a Kretschmann configuration. To compute the reflectance of the multilayer system, we employ the transfer matrix formalism, which relates the tangential components of the electromagnetic fields across each interface. For a uniaxial layer of thickness d, the transfer matrices written in the basis of the tangential field components (H_y, E_x), for TM and TE polarizations are given by:

$$T_{\mathrm{TM}}=\begin{pmatrix} cos(k_{2z}^{(e)}d) & i\frac{\omega\varepsilon_{xx}}{ck_{2z}^{(e)}}sin(k_{2z}^{(e)}d) \\ i\frac{k_{2z}^{(e)}c}{\omega\varepsilon_{xx}}sin(k_{2z}^{(e)}d) & cos(k_{2z}^{(e)}d) \end{pmatrix};\ T_{\mathrm{TE}}=\begin{pmatrix} cos(k_{2z}^{(o)}d) & i\frac{\omega}{ck_{2z}^{(o)}}sin(k_{2z}^{(o)}d) \\ i\frac{k_{2z}^{(o)}c}{\omega}sin(k_{2z}^{(o)}d) & cos(k_{2z}^{(o)}d) \end{pmatrix}$$

where $k_{2z}^{(e)}$ and $k_{2z}^{(o)}$ are the longitudinal wavevector components for the extraordinary (TM) and ordinary (TE) modes in the uniaxial film, respectively. These are defined as:

$$k_{2,z}^{(e)^2}=\frac{\varepsilon_{xx}}{\varepsilon_{zz}}\left(\varepsilon_{zz}\frac{\omega^2}{c^2}-q^2\right);\ k_{2,z}^{(o)^2}=\varepsilon_{xx}\frac{\omega^2}{c^2}-q^2$$

where the in-plane (transverse) wavevector is $q=\sqrt{\varepsilon_1}(\omega/c)\ sin\ \theta_i$, with θ_i denoting the angle of incidence at the prism/J-aggregate interface. These matrices allow us to propagate the electromagnetic fields through the excitonic layer, from z=0 to z=d.

The total transfer matrix of the system can then be used to derive the transmission ($\hat{t}$) and reflection ($\hat{r}$) coefficients for each polarization. They are given by:

$$\hat{t}_{\mathrm{TM}}=\frac{2}{T_{11}^{-1}+\frac{ck_{3z}}{\omega\varepsilon_3}T_{12}^{-1}+\frac{\omega\varepsilon_1}{ck_{1z}}T_{21}^{-1}+\frac{k_{3z}\varepsilon_1}{k_{1z}\varepsilon_3}T_{22}^{-1}};\ \hat{t}_{\mathrm{TE}}=\frac{2}{T_{11}^{-1}+\frac{ck_{3z}}{\omega}T_{12}^{-1}+\frac{\omega}{ck_{1z}}T_{21}^{-1}+\frac{k_{3z}}{k_{1z}}T_{22}^{-1}}$$

$$\hat{r}_{\mathrm{TM}}=\left(T_{11}^{-1}+\frac{ck_{3z}}{\varepsilon_3\omega}T_{12}^{-1}\right)\hat{t}-1;\ \hat{r}_{\mathrm{TE}}=\left(T_{11}^{-1}+\frac{ck_{3z}}{\omega}T_{12}^{-1}\right)\hat{t}-1$$

where media 1 and 3 correspond to the prism and air, respectively. The longitudinal wavevector components in these media are:

$$k_{1(3)z}=\sqrt{\varepsilon_{1(3)}\frac{\omega^2}{c^2}-q^2}$$

and T_{ij}^{-1} denotes the (i, j) element of the inverse transfer matrix associated with the corresponding polarization. Finally, the reflectance spectra for both polarizations are obtained as:

$$R_{TM}=|\hat{r}_{TM}|^2;\ R_{TE}=|\hat{r}_{TE}|^2$$

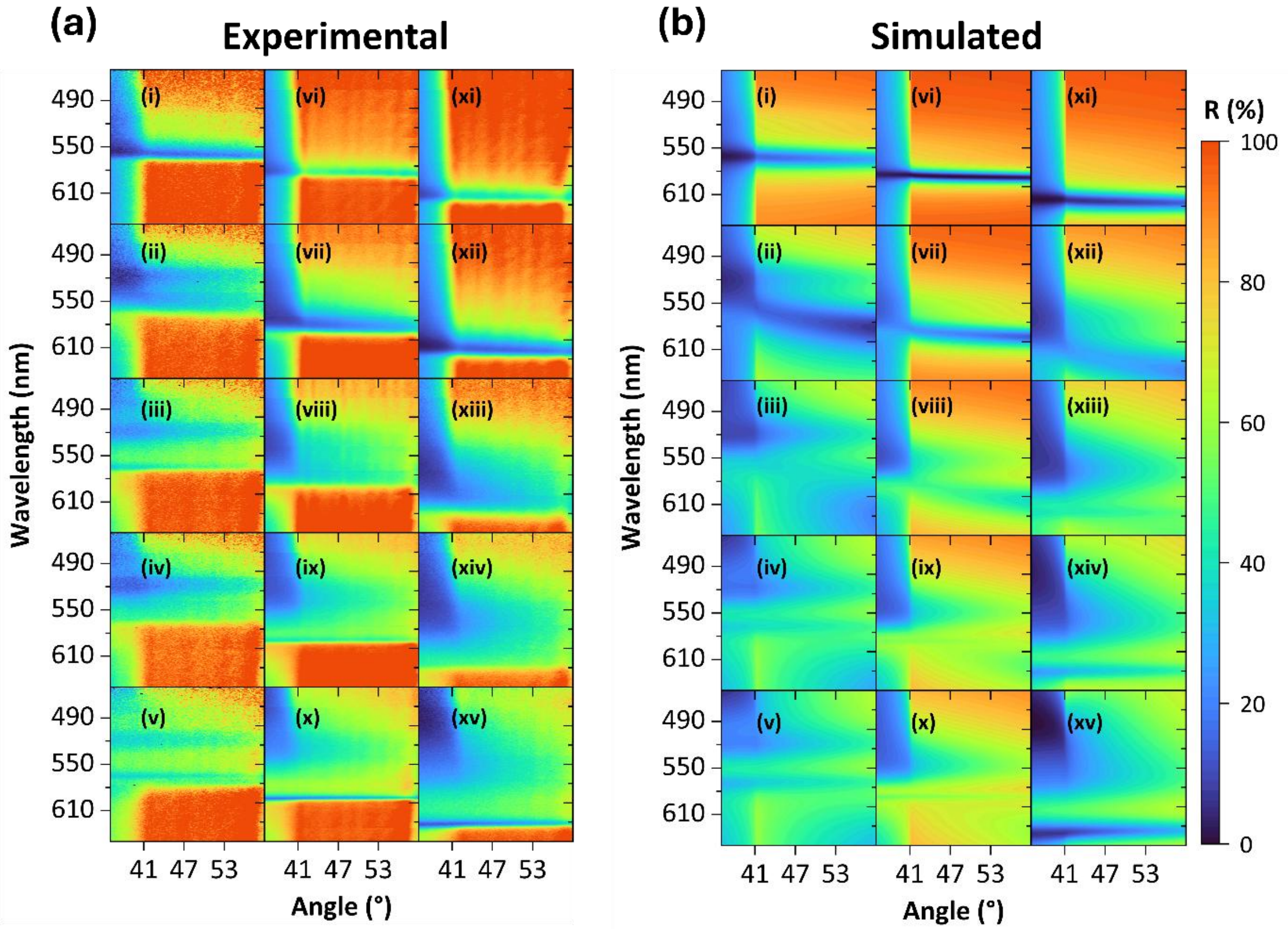

Figure S8. (a) Experimental and **(b)** simulated TE-polarized angular reflectance of J-aggregate thin films under surface waves coupling conditions for increasing deposition cycles. (i) 1, (ii) 5 (iii) 10, (iv) 15, and (v) 17 j560 depositions; (vi) 2, (vii) 5, (viii) 10, (ix) 15, and (x) 20 j590 depositions; (xi) 1, (xii) 5, (xiii) 10, (xiv) 15, and (xv) 20 j620 depositions. Theoretical modelling was performed using J-aggregate bulk film models, using the anisotropic permittivity obtained by ellipsometry and thicknesses estimated by AFM.

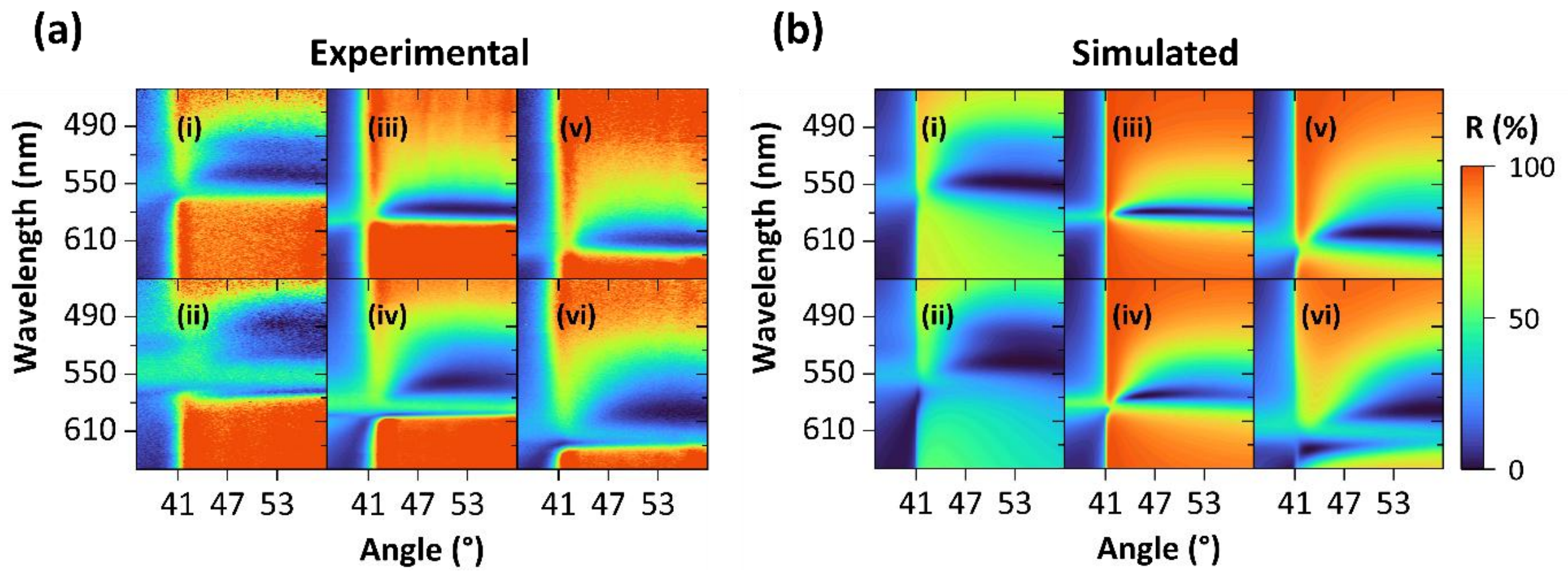

Figure S9. (a) Experimental and **(b)** simulated TM-polarized angular reflectance of J-aggregate thin films under surface waves coupling conditions for increasing deposition cycles. (i) 10 and (ii) 17 j560 depositions; (iii) 10 and (iv) 20 j590 depositions; (v) 10 and (vi) 20 j620 depositions. Theoretical modelling was performed using J-aggregate bulk film models, using the anisotropic permittivity obtained by ellipsometry and thicknesses estimated by AFM.

Section S4. Electric Field Profiles

To plot the field profile in the Kretschmann configuration, we first write the expressions for the electromagnetic fields in each region. Considering the experimental setup described in detail on section 4.9 of the main manuscript, in the oil-substrate region, located at $z<0$, the field is expressed as the sum of incident and reflected propagating waves:

$$\vec{E}_1 = \left[\left(E_{1,x}^{(i)}, 0, E_{1,z}^{(i)}\right)e^{ik_{1z}z} + \left(E_{1,x}^{(r)}, 0, E_{1,z}^{(r)}\right)e^{-ik_{1z}z}\right]e^{i(qx-\omega t)};$$
$$\vec{H}_1 = \left[\left(0, H_{1,y}^{(i)}, 0\right)e^{ik_{1z}z} + \left(0, H_{1,y}^{(r)}, 0\right)e^{-ik_{1z}z}\right]e^{i(qx-\omega t)}.$$

where $E^{(i)}$ and $E^{(r)}$ mean incident and reflected electric field. In the J-aggregate film, located at $0<z<d$, the field is a superposition of decaying and growing evanescent waves. Thus, we write:

$$\vec{E}_2 = \left[\left(E_{2,x}^{(+)}, 0, E_{2,z}^{(+)}\right)e^{\kappa_{2z}z} + \left(E_{2,x}^{(-)}, 0, E_{2,z}^{(-)}\right)e^{-\kappa_{2z}z}\right]e^{i(qx-\omega t)};$$
$$\vec{H}_2 = \left[\left(0, H_{2,y}^{(+)}, 0\right)e^{\kappa_{2z}z} + \left(0, H_{2,y}^{(-)}, 0\right)e^{-\kappa_{2z}z}\right]e^{i(qx-\omega t)}.$$

where $E^{(+)}$ and $E^{(-)}$ mean growing and evanescent waves. Finally, in the air region ($z>d$), the field is once again evanescent and can be written as:

$$\vec{E}_3 = \left(E_{3,x}, 0, E_{3,z}\right)e^{-\kappa_{3z}z}e^{i(qx-\omega t)};$$
$$\vec{H}_3 = \left(0, H_{3,y}, 0\right)e^{-\kappa_{3z}z}e^{i(qx-\omega t)}.$$

The wavevectors of the previous expressions are defined as:

$$k_{1z} = \sqrt{\varepsilon_1\frac{\omega^2}{c^2} - q^2};\ \kappa_{2z} = \sqrt{\frac{\varepsilon_{xx}}{\varepsilon_{zz}}\left(q^2 - \varepsilon_{zz}\frac{\omega^2}{c^2}\right)};\ \kappa_{3z} = \sqrt{q^2 - \varepsilon_3\frac{\omega^2}{c^2}}.$$

To compute the field profiles, we need first to relate the several field amplitudes. To do that we can apply boundary conditions. At z=0 we obtain:

$$E_{1x}^{(i)}+E_{1x}^{(r)}=E_{2x}^{(+)}+E_{2x}^{(-)}\,;$$
$$H_{1y}^{(i)}+H_{1y}^{(r)}=H_{2y}^{(+)}+H_{2y}^{(-)}\,,$$

and at z=d we get:

$$E_{2x}^{(+)}e^{\kappa_{2z}d}+E_{2x}^{(-)}e^{-\kappa_{2z}d}=E_{3x}e^{-\kappa_{3z}d}\,;$$
$$H_{2y}^{(+)}e^{\kappa_{2z}d}+H_{2y}^{(-)}e^{-\kappa_{2z}d}=H_{3y}e^{-\kappa_{3z}d}\,.$$

By using Ampère's law:

$$\vec{\nabla}\times\vec{H}=\frac{1}{c}\frac{\partial\vec{D}}{\partial t}\,,$$

we can relate the magnetic and electric field amplitudes which lead us to:

$$H_{1y}^{(i)}=\frac{\omega\varepsilon_1}{ck_{1z}}E_{1x}^{(i)}\,;\;H_{1y}^{(r)}=-\frac{\omega\varepsilon_1}{ck_{1z}}E_{1x}^{(r)}\,;$$
$$H_{2y}^{(+)}=\frac{i\omega\varepsilon_{xx}}{c\kappa_{2z}}E_{2x}^{(+)}\,;\;H_{2y}^{(-)}=-\frac{i\omega\varepsilon_{xx}}{c\kappa_{2z}}E_{2x}^{(-)}\,;H_{3y}=\frac{i\omega\varepsilon_3}{c\kappa_{3z}}E_{3x}\,.$$

Therefore, it allows us to write the following system of equations:

$$\begin{pmatrix}1 & -1 & -1 & 0\\ -\dfrac{\varepsilon_1}{k_{1z}} & -\dfrac{i\varepsilon_{xx}}{\kappa_{2z}} & \dfrac{i\varepsilon_{xx}}{\kappa_{2z}} & 0\\ 0 & e^{\kappa_{2z}d} & e^{-\kappa_{2z}d} & -e^{-\kappa_{3z}d}\\ 0 & \dfrac{\varepsilon_{xx}}{\kappa_{2z}}e^{\kappa_{2z}d} & -\dfrac{\varepsilon_{xx}}{\kappa_{2z}}e^{-\kappa_{2z}d} & \dfrac{\varepsilon_3}{\kappa_{3z}}e^{-\kappa_{3z}d}\end{pmatrix}\begin{pmatrix}E_{1x}^{(r)}/E_{1x}^{(i)}\\ E_{2x}^{(+)}/E_{1x}^{(i)}\\ E_{2x}^{(-)}/E_{1x}^{(i)}\\ E_{3x}/E_{1x}^{(i)}\end{pmatrix}=\begin{pmatrix}-1\\ -\dfrac{\varepsilon_1}{k_{1z}}\\ 0\\ 0\end{pmatrix}\Rightarrow M\begin{pmatrix}E_{1x}^{(r)}/E_{1x}^{(i)}\\ E_{2x}^{(+)}/E_{1x}^{(i)}\\ E_{2x}^{(-)}/E_{1x}^{(i)}\\ E_{3x}/E_{1x}^{(i)}\end{pmatrix}=A$$

This enables us to compute all the field components as function of the incident field $E_{1x}^{(i)}$. For the reflected field we have:

$$\frac{E_{1x}^{(r)}}{E_{1x}^{(i)}}=\frac{\det(M_1)}{\det(M)};\;\frac{E_{2x}^{(+)}}{E_{1x}^{(i)}}=\frac{\det(M_2)}{\det(M)};\;\frac{E_{2x}^{(-)}}{E_{1x}^{(i)}}=\frac{\det(M_3)}{\det(M)};\;\frac{E_{3x}}{E_{1x}^{(i)}}=\frac{\det(M_4)}{\det(M)}$$

where M_i is the matrix obtained by replacing the i-th column of M with the vector A.

To fully define the electric field, we can also use Ampere's law to compare the z and x amplitudes. We obtain:

$$E_{1z}^{(i)}=-\frac{q}{\kappa_{1z}}E_{1x}^{(i)}\,;\;E_{1z}^{(r)}=\frac{q}{\kappa_{1z}}E_{1x}^{(r)}\,;$$
$$E_{2z}^{(+)}=-\frac{i\varepsilon_{xx}q}{\varepsilon_{zz}\kappa_{2z}}E_{2x}^{(+)}\,;\;E_{2z}^{(-)}=\frac{i\varepsilon_{xx}q}{\varepsilon_{zz}\kappa_{2z}}E_{2x}^{(-)}\,;\;E_{3z}=\frac{iq}{\kappa_{3z}}E_{3x}\,.$$

Therefore, we finally write the electric field as:

$$\vec{E}_1=\left[\left(E^{(i)}_{1,x},0,-\frac{q}{k_{1z}}E^{(i)}_{1x}\right)e^{ik_{1z}z}+\left(E^{(r)}_{1,x},0,\frac{q}{k_{1z}}E^{(r)}_{1x}\right)e^{-ik_{1z}z}\right]e^{i(qx-\omega t)};$$

$$\vec{E}_2=\left[\left(E^{(+)}_{2,x},0,-\frac{i\varepsilon_{xx}q}{\varepsilon_{zz}\kappa_{2z}}E^{(+)}_{2x}\right)e^{\kappa_{2z}z}+\left(E^{(-)}_{2,x},0,\frac{i\varepsilon_{xx}q}{\varepsilon_{zz}\kappa_{2z}}E^{(-)}_{2x}\right)e^{-\kappa_{2z}z}\right]e^{i(qx-\omega t)};$$

$$\vec{E}_3=\left(E_{3,x},0,\frac{iq}{\kappa_{3z}}E_{3x}\right)e^{-\kappa_{3z}z}e^{i(qx-\omega t)}.$$

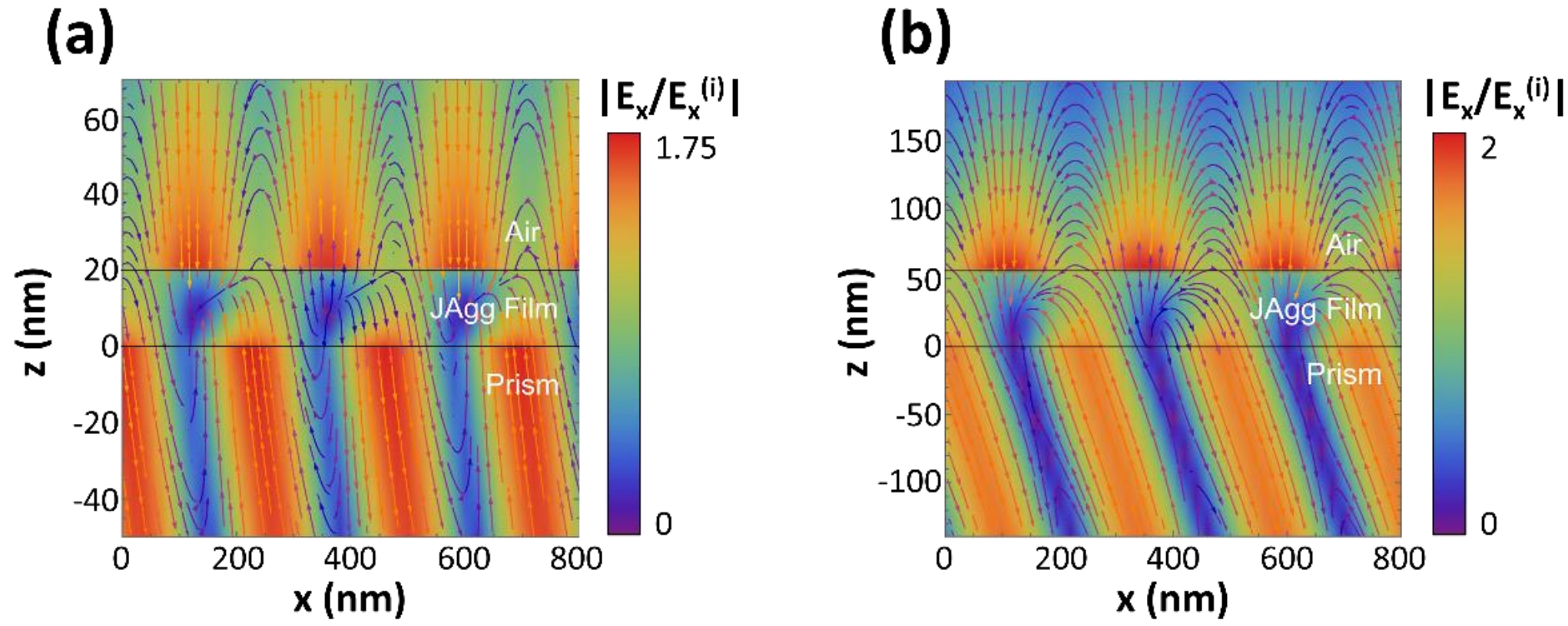

Figure S10. Two-dimensional colourmaps of the modulus of E_x normalised by the incident electric field E(i) in a cross-section of a thin film with 10 depositions with an incident angle of 55° for **(a)** j590 with λ = 589 nm and for **(b)** j620 with λ = 619 nm. Displayed arrows correspond to the electric field vector $\vec{E} = (E_x, E_z)$.

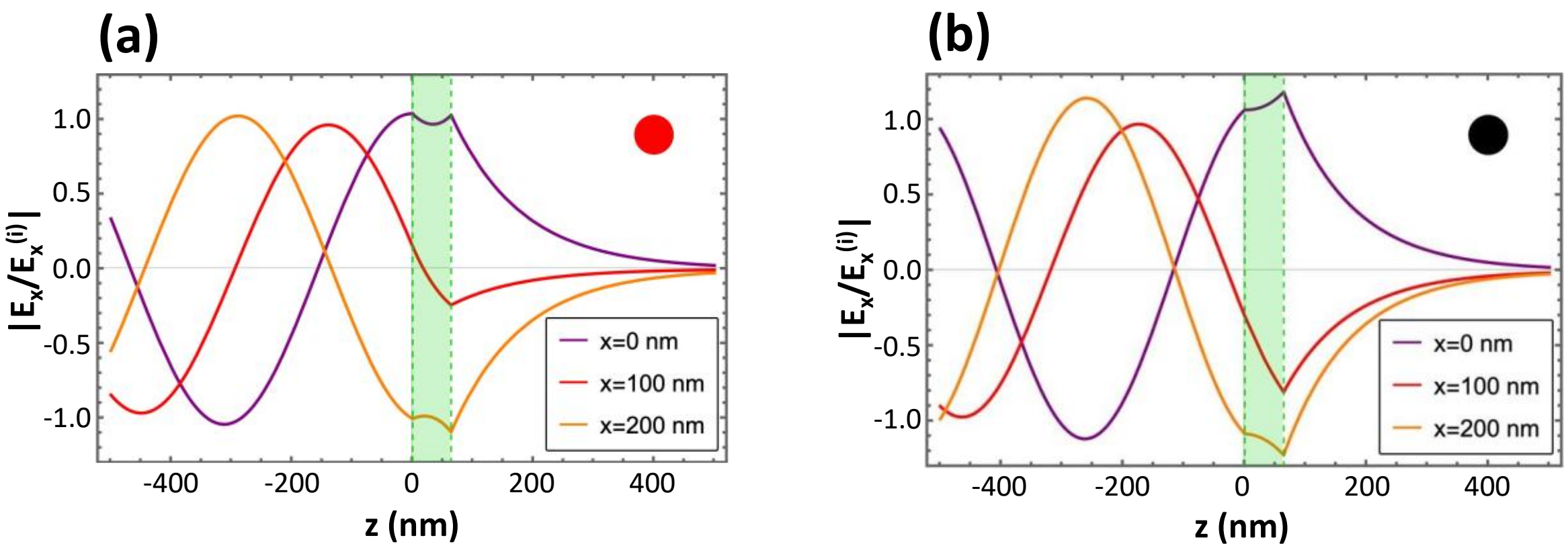

Figure S11. z-direction profiles for different x values of the modulus of E_x normalised by the incident electric field E(i) in a cross-section of a j560 thin film with 10 depositions with an incident angle of 55° for **(a)** λ = 540 nm (red line dot in Figure 5b.iii) and for **(b)** λ = 505 nm (black dot in Figure 5b.iii).

Table S3. Magnitude of the wavevector z-component for a j560 thin film with 15 depositions with incident angle θ_i = 55° for the wavelengths corresponding to the TMM-simulated reflectance minima of the two J-aggregate conformations.

λ (nm)	k_{1z} (μm^{-1})	k_{2z} (μm^{-1})	k_{3z} (μm^{-1})
505 (J^2_{560})	10.83	6.95 - 4.85i	9.19
540 (J^1_{560})	10.13	11.39 - 4.46i	8.60

Section S4. Methods

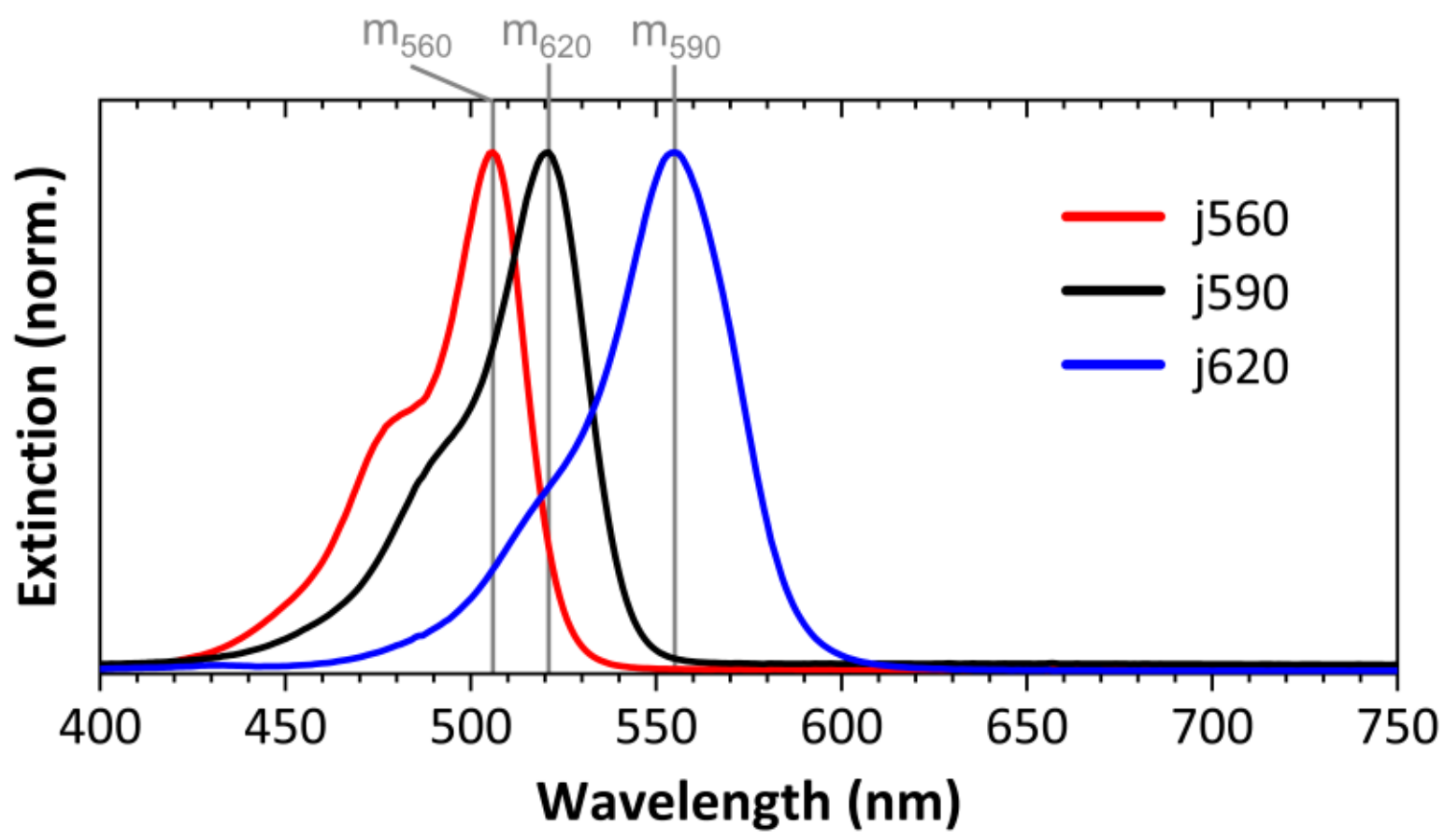

Figure S12. Normalized extinction spectra of different carbocyanine dyes in ethanol. Vertical lines mark the peak maxima: m_{560} at 506 nm, m_{590} at 521 nm, and m_{620} at 555 nm. Here, m denotes the monomer peaks.

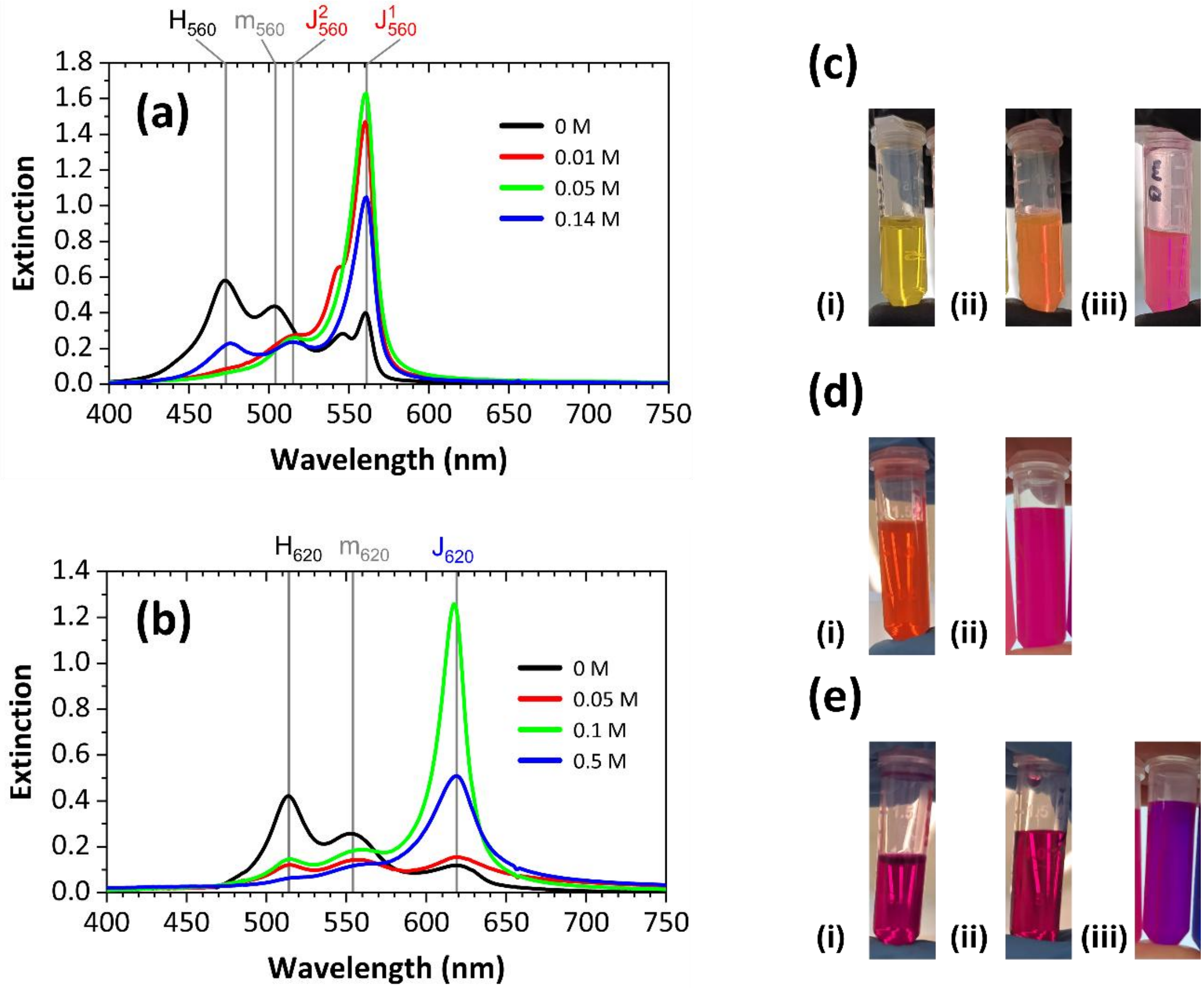

Figure S13. (a-b) Extinction spectra of aqueous j560 **(a)** and j620 **(b)** solutions at varying NaCl concentrations, as indicated. The monomer concentration was 100 µM. Vertical lines mark the peak maxima: H_{560} at 473, m_{560} at 506, J^2_{560} at 515, J^1_{560} at 561, H_{620} at 513, m_{620} at 555, and J_{620} at 619 nm**.** Here, H denotes H-aggregate peaks, J denotes J-aggregate peak, and m denotes the monomer peaks. **(c-e)** Photographs of dissolved carbocyanines: **(c)** j560, **(d)** j590, and **(e)** j620 in (i) ethanol, (ii) milli-Q water, and (iii) milli-Q water with optimal ionic strength (NaCl) to induce J-aggregate formation.
